# Novel gene function revealed by mouse mutagenesis screens for models of age-related disease

**DOI:** 10.1038/ncomms12444

**Published:** 2016-08-18

**Authors:** Paul K. Potter, Michael R. Bowl, Prashanthini Jeyarajan, Laura Wisby, Andrew Blease, Michelle E. Goldsworthy, Michelle M. Simon, Simon Greenaway, Vincent Michel, Alun Barnard, Carlos Aguilar, Thomas Agnew, Gareth Banks, Andrew Blake, Lauren Chessum, Joanne Dorning, Sara Falcone, Laurence Goosey, Shelley Harris, Andy Haynes, Ines Heise, Rosie Hillier, Tertius Hough, Angela Hoslin, Marie Hutchison, Ruairidh King, Saumya Kumar, Heena V. Lad, Gemma Law, Robert E. MacLaren, Susan Morse, Thomas Nicol, Andrew Parker, Karen Pickford, Siddharth Sethi, Becky Starbuck, Femke Stelma, Michael Cheeseman, Sally H. Cross, Russell G. Foster, Ian J. Jackson, Stuart N. Peirson, Rajesh V. Thakker, Tonia Vincent, Cheryl Scudamore, Sara Wells, Aziz El-Amraoui, Christine Petit, Abraham Acevedo-Arozena, Patrick M. Nolan, Roger Cox, Anne-Marie Mallon, Steve D. M. Brown

**Affiliations:** 1 MRC Harwell Institute, Mammalian Genetics Unit, Harwell Campus, Oxfordshire OX11 0RD, UK; 2 Génétique et Physiologie de l'Audition, Institut Pasteur, INSERM UMR-S 1120, Sorbonne Universités, UPMC Univ Paris 06, Collège de France, 25 rue Dr Roux, Paris 75015, France; 3 The Nuffield Laboratory of Ophthalmology & NIHR Oxford Biomedical Research Centre, University of Oxford, Oxford OX3 9DU, UK; 4 The Roslin Institute and Royal (Dick) School of Veterinary Studies, University of Edinburgh, Edinburgh EH25 9RG, UK; 5 MRC Human Genetics Unit, Institute of Genetics and Molecular Medicine, University of Edinburgh, Western General Hospital, Edinburgh EH4 2XU, UK; 6 Nuffield Department of Clinical Neurosciences (Nuffield Laboratory of Ophthalmology), John Radcliffe Hospital, University of Oxford, Oxford OX3 9DU, UK; 7 Oxford Centre for Diabetes, Endocrinology and Metabolism, University of Oxford, Churchill Hospital, Oxford OX3 7LJ, UK; 8 Kennedy Institute of Rheumatology, Nuffield Department of Orthopaedics, Rheumatology and Musculoskeletal Sciences, University of Oxford, Oxford OX3 7FY, UK

## Abstract

Determining the genetic bases of age-related disease remains a major challenge requiring a spectrum of approaches from human and clinical genetics to the utilization of model organism studies. Here we report a large-scale genetic screen in mice employing a phenotype-driven discovery platform to identify mutations resulting in age-related disease, both late-onset and progressive. We have utilized *N*-ethyl-*N*-nitrosourea mutagenesis to generate pedigrees of mutagenized mice that were subject to recurrent screens for mutant phenotypes as the mice aged. In total, we identify 105 distinct mutant lines from 157 pedigrees analysed, out of which 27 are late-onset phenotypes across a range of physiological systems. Using whole-genome sequencing we uncover the underlying genes for 44 of these mutant phenotypes, including 12 late-onset phenotypes. These genes reveal a number of novel pathways involved with age-related disease. We illustrate our findings by the recovery and characterization of a novel mouse model of age-related hearing loss.

## Abstract (web summary)

Random mutagenesis can uncover novel genes involved in phenotypic traits. Here the authors perform a large-scale phenotypic screen on over 100 mouse strains generated by ENU mutagenesis to identify mice with age-related diseases, which they attribute to specific mutations revealed by whole-genome sequencing.

## Graphical abstract

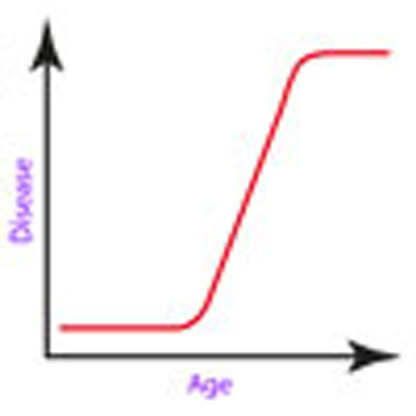

The increasing incidence and burden of diseases of ageing highlights the need to identify the genetic determinants of age-related disease. A wide variety of diseases are associated with ageing including neurodegenerative disorders, diabetes, cancer, kidney disease and cardiovascular disease (reviewed in ref. 1). However, the genetic basis for many of these diseases remains poorly understood. A proven methodology to identify novel genes and pathways associated with disease, which has been employed successfully across a wide range of physiological systems, is *N*-ethyl-*N*-nitrosourea (ENU) -based phenotype-driven screening^2^,^3^. The principle involves inducing random point mutations throughout the murine genome with ENU and then screening offspring for phenotypes of interest^4^. Mapping and subsequent candidate gene, or more recently whole genome^5^,^6^, sequencing then uncovers the causative mutation. Phenotype-driven screens have been successful in identifying novel genes and alleles across a wide range of phenotypic areas^4^. In some cases an additional challenge is required to reveal a phenotype, whereby the effect of the challenge and underlying mutation combine to result in a phenotype. The success of this strategy is exemplified by gene function revealed by immunological challenges^7^,^8^.

Age is an important challenge for incorporation in phenotype-driven screens. Many factors combine to contribute to an ageing physiology, including mitochondrial dysfunction^9^ and cellular senescence^10^, which may interact with genetic variants to result in age-related phenotypes. Here we describe our initial findings from a screen designed to reveal mutations that result in age-related disease. We have adopted a phenotype-driven, forward genetic approach utilizing ENU mutagenesis accompanied by recurrent phenotype screens of aged mutant mice. This has allowed us to identify phenotypes occurring late in life resulting from specific ENU-induced mutations. Such mutants identify genes and pathways that contribute to age-related disease and allows us to characterize the interactions of these pathways with an ageing physiology that result specifically in an age-related phenotype. We demonstrate that this is a successful approach for identifying mouse mutants with late-onset phenotypes, and importantly find that these mutant models reveal a significant number of novel genes and pathways involved with such diseases.

## Results

### Pedigree production

We established a large-scale mouse screen for recessive mutations causing age-related disease incorporating an ENU mutagenesis^4^ and phenotype-driven approach. We screened G_3_ mice in large pedigrees of up to 100 mice^4^ (Supplementary Fig. 1a). Briefly, male C57BL/6J mice were mutagenized with ENU and mated to C3H.Pde6b+ mice^11^ to generate G_1_ founder males. G_1_ males were subsequently bred to C3H.Pde6b+ mice to generate G_2_ offspring. Next, large G_3_ pedigrees of at around 100 individuals were produced by two rounds of mating of the founder G_1_ male to ⩾8 G_2_ female offspring. Large pedigrees ensured sufficient homozygous G_3_ individuals were available to map a phenotype directly from the G_3_ cohort. Each G_3_ pedigree was generated as two cohorts, 2–3 months apart. Across an entire G_3_ pedigree comprising some 100 mice, we might expect to identify on average 12 affected individuals homozygous for an individual recessive mutation inherited from the G_1_ founder male. The mixed genetic background assisted the mapping of mutations underlying the affected individuals identified in the G_3_ pedigrees.

Wild-type C3H strains exhibit retinal degeneration, due to a recessive mutation in the *Pde6b* gene (*Pde6b*^*rd1*^), and hence we employed the line, C3H.Pde6b+ (ref. 11), congenic for the BALB/c region encompassing *Pde6b* and which does not exhibit retinal degeneration enabling screening for visual abnormalities. C57BL/6J also carries a recessive mutation in the *Cdh23* gene resulting in age-related hearing loss (*Cdh23*^*ahl*^) (ref. 12). Therefore, in our protocol G_2_ female mice were genotyped for the *Cdh23*^*ahl*^ allele and only offspring from G_2_ females wild-type for *Cdh23* were used for auditory assessment.

### Phenotyping pipeline and mutant detection

We developed and implemented a high-throughput phenotyping pipeline to examine the G_3_ mice generated in the ageing screen (Table 1 and summarized in Supplementary Fig. 1b). This incorporated many of the established standard operating procedures that have been utilized in the International Mouse Phenotyping Consortium (IMPC, www.impc.org) phenotyping pipeline^13^. The IMPC has implemented IMPReSS (www.mousephenotype.org/impress), a database of standardized phenotyping pipelines that encompasses a large number of disease systems and ensures data reproducibility and integrity. The design of the pipeline considered carefully the timing and order of the phenotyping tests to ensure that we minimized the impact of individual tests on other phenotype outcomes. For each G_3_ pedigree the production of dual cohorts provided an opportunity to explore in more detail phenotypes detected in the first cohort and to add complementary phenotype tests. The scale of the screen precluded a complete pathological examination of tissues from all G_3_ mice produced, but this was carried out on selected mice to assist in the confirmation or determination of a phenotype. We have analysed 157 pedigrees to date, out of which 134 are complete and 23 are currently active (Table 2). The average pedigree size was 102 mice and a total of 13,601 G_3_ animals have been generated and phenotyped from the 157 pedigrees.

### Phenotype detection and MouseBook

We devised a high-throughput automated phenotype-detection strategy, alongside a resource to visualize the phenotyping data. We defined a mutant line as one where multiple affected G_3_ mice (⩾3) in a pedigree display very similar phenotypes. All raw data from the phenotyping platforms was initially captured into local laboratory information management systems according to the data standards defined in the IMPReSS database. Following quality control procedures, a step-wise strategy was devised to detect outliers from temporal data in ENU pedigrees. Briefly, we used a reference range approach that established percentile values for the entire data set that provides high and low critical values, approximately equating to ±2 s.d. Parameter values outside of these critical values for three or more animals in a pedigree led to a phenodeviancy call. Subsequently, phenodeviant mice were annotated with the appropriate phenotype (MP term) as specified by IMPReSS. All raw data and automatically annotated phenotype data are available via the publically accessible data portal MouseBook (http://www.mousebook.org/).

To assess the false positive rate (FPR) of the reference range method, we calculated the FPR of wild-type C57BL/6NTac animal data from the IMPC project as a test data set. IMPC uses similar phenotype procedures to the ageing project. We then modelled the ageing pedigrees by sampling random IMPC baseline animals to produce a simulated pedigree and employed the reference range method on each procedure. Wild-type animals should not be called as outliers therefore any positive calls from the method are classed as false positive calls. Using simulated pedigrees, we detected between 0.58 and 1.22% phenodeviance, suggesting a low average FPR of 0.71% across the procedures examined (Supplementary Table 1).

Categorical data, such as X-ray abnormalities, were scored as potential mutants when observed in multiple affected G_3_ mice (⩾3) in a pedigree. We also, on occasion, considered mice as phenodeviant when outliers were <2 s.d. from the mean but where deviations were sustained over multiple time points or, alternatively, additional phenotyping data confirmed a phenotype was present. Each putative mutant was confirmed by one or all of the following; appropriate statistical analyses of primary phenotyping data, comparison of data from other relevant phenotypic tests, inheritance testing and mapping data.

### Mutant lines

**Figure 1:**
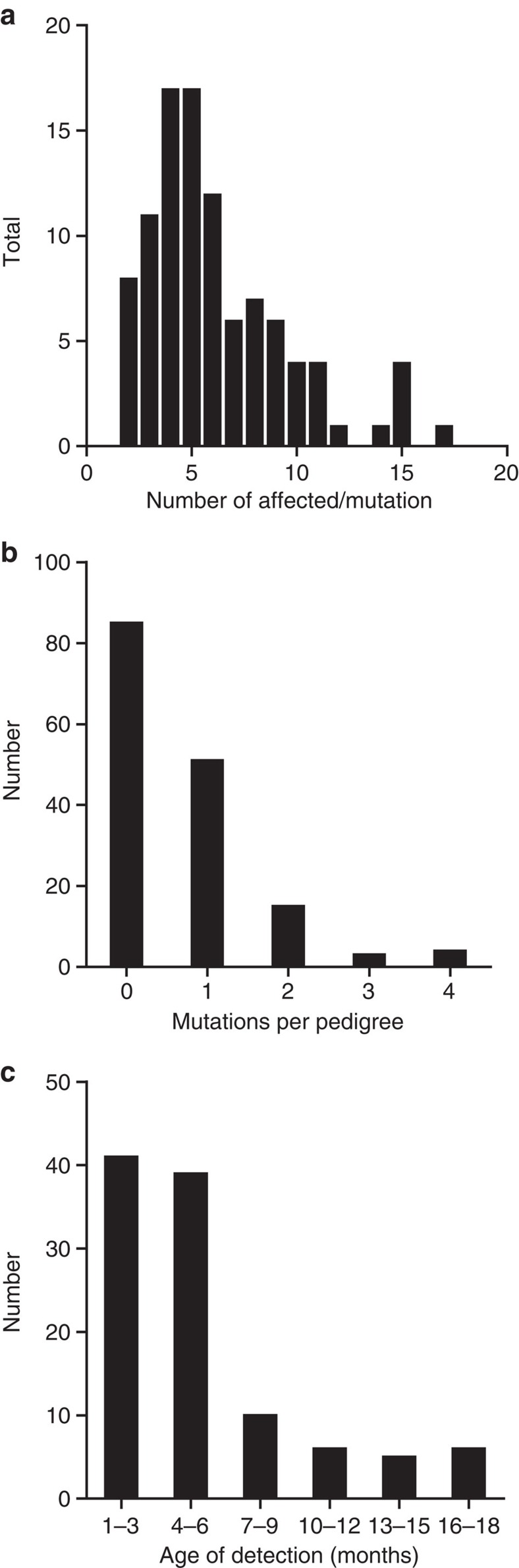
Summary statistics for mutation discovery in the Harwell ageing Screen. (**a**) The distribution of the numbers of affected mice observed for each mutation within the G_3_ pedigrees. For lines with low numbers of affected mice, putative mutations were confirmed by inheritance testing and/or cloning of the underlying mutation. (**b**) The distribution of the number of mutations identified per pedigree from a total of 105 mutant lines identified from a total of 157 pedigrees. (**c**) The distribution of mutation discovery over time. The time point of the initial identification of an individual phenotype is represented here for a total of 105 mutant lines identified in the screen to date. Where mutants were outliers in more than one phenotypic test the time point of the first test to reveal an abnormality is shown.

In total, 105 distinct mutant lines were identified in 72 out of 157 pedigrees completed or currently undergoing analysis (Supplementary Table 2). The mean number of affected G_3_ mice for each line was 6.1, with a range from 2 to 17 (Fig. 1a). Figure 1b demonstrates the distribution of the number of mutations detected per pedigree. Some pedigrees had multiple segregating mutations, which may reflect the size of the pedigrees and the inherent chance of detecting multiple affected mice for distinct genetic lesions.

Abnormal phenotypes were identified throughout the lifespan of the pedigrees (Fig. 1c). Visible anomalies such as coat colour variations, dysmorphologies and extreme behavioural abnormalities were often detected during initial husbandry practices early in the pipeline before scheduled screens took place. Between 3 and 6 months the first round of tests uncovered a large number of additional phenotypes. Most importantly, of the 105 mutant lines identified, 27 were only detected after 6 months (Fig. 1c).

### Mapping and mutation detection

Individual mutations were mapped utilizing the Illumina Medium Density SNP mapping panel, which is informative at over 900 single-nucleotide polymorphisms (SNPs) for the C3H.Pde6b+ and C57BL/6J strains. The genotypes of affected animals were compared with those of unaffected individuals from the same pedigree, generally resulting in a map location of around ∼20 Mb. Following mapping, we carried out whole-genome sequencing (WGS) of the G_1_ founder male, or, on occasion, an affected G_3_. A local database was developed to manage the WGS data from G_1_ mice. All WGS data was analysed through a standard next-generation sequencing (NGS) pipeline. The MouseBook portal holds the complete range of ENU mutations identified in G_1_ mice (noncoding and coding mutations) and within noncoding regulatory regions (such as lincRNAs, miRNAs and promoter regions) annotated from WGS analysis pipeline. This archive includes all ENU mutations identified during NGS, including all incidental mutations that have not been associated with a phenotype. This data set enabled rapid identification of all mutations segregating within the sequenced pedigrees and this information, combined with the map location of the phenotype, allowed us to determine the causative ENU-induced genetic lesion with high confidence.

For the 105 lines identified we have currently mapped 81. WGS of 28 G_1_ and 8 G_3_ mice identified the gene lesion underlying the observed phenotype in 44 mutant lines (Table 2 and Supplementary Table 2). For individual pedigrees that contained multiple segregating phenotypes (Fig. 1b) we were able to identify several causative mutations from a single-G_1_ sequence. Some obvious candidate genes are undergoing individual gene sequencing and some phenotypes were not of sufficient impact to warrant WGS. For three of the mapped mutations analysed (all early phenotypes) we failed to uncover a coding lesion, and these may represent functional changes in noncoding DNA. One coding lesion was identified as an intracisternal A-particle insertion that appears to have occurred spontaneously on the C3H.Pde6b+ background pedigree MPC-59, disrupting the *Mcr1* gene and resulting in a yellow coat colour (Supplementary Table 2). Out of the 27 ageing phenotypes, 23 have been mapped and for 12 late-onset mutant lines we have identified and confirmed the underlying mutation (Table 3).

### Genetic and phenotypic analysis of mutants

**Figure 2:**
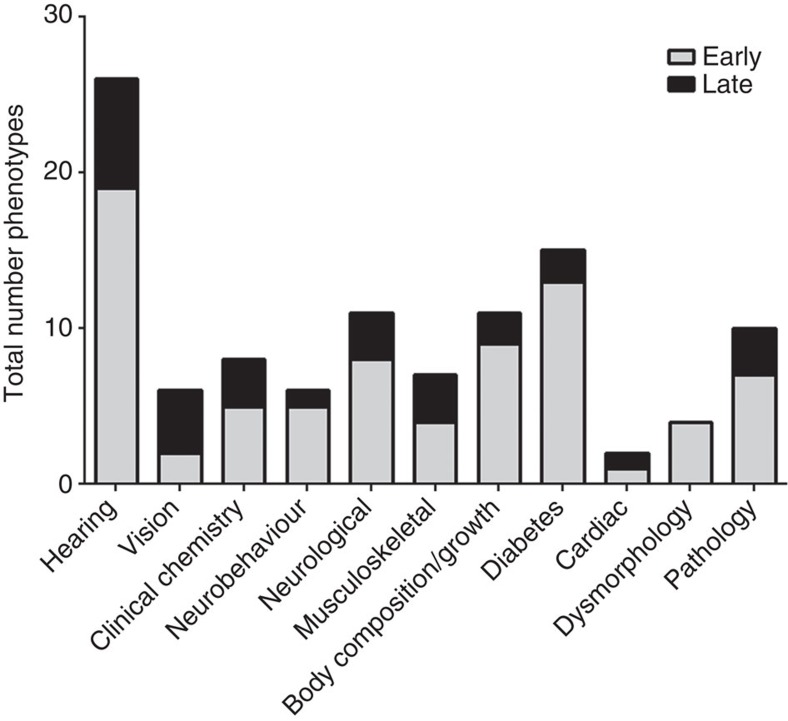
The distribution of early and late phenotypes across a variety of disease and biological categories. A histogram of the distribution of early and late phenotypes across different phenotypic categories. Late phenotypes are defined as those phenotypes where affected mice were identified at 7 months or later. Note that we catalogue the number of individual ‘phenotypes' in each disease or biological category, and that individual mutant lines may demonstrate multiple phenotypes.

We carried out a high-level ontological analysis of the distribution of phenotypes across disease areas investigated by our phenotyping pipeline. The total number of phenotypes observed across different phenotypic areas is shown in Fig. 2. Where a single line exhibited more than one phenotype resulting from the pleiotropic effects of the underlying mutation we have listed all the phenotypes detected. In the majority of phenotypic areas we have identified late-onset phenotypes, but for some categories we have uncovered no late-onset models. For example, dysmorphology mutants are overt abnormalities normally associated with developmental defects, and hence are unlikely to develop late in life.

Table 3 summarizes the genes that have been identified for late-onset phenotypes. In each case, we have either uncovered a gene for which there was no prior functional information, or alternatively, we have assigned novel functionality to a gene with known functions. For these mutants there is only a single medium or high-confidence coding mutation in the minimal mapping region and for laminin alpha 5 (*Lama5*) and tryptophanyl tRNA synthetase 2, mitochondrial (*Wars2*) (pedigrees MPC-205 and MPC-151, respectively) we have carried out complementation studies using a knockout (KO) allele to confirm these mutations as the causative allele (Table 3). In most cases, we also have identified functional alterations associated with the mutation that could account for the observed phenotype but, as these studies are ongoing, in several cases the causal link between mutation and phenotype are yet to be proven conclusively. Several examples in diverse biological areas serve to illustrate this powerful discovery platform for models of age-related disease.

We identified a missense mutation (E884G) in the *Lama5* gene that results in a progressive nephrotic syndrome. G_3_ mice were identified at 6 months of age with elevated plasma urea and creatinine levels, and with reduced plasma albumin (homozygotes, *n*=6, versus wild-type, *n*=33, Urea: 32.9±19.9 versus 5.9±1.1 mmol l^−1^, *P*<0.05, creatinine: 50.4±37.1 versus 11.45±3.6 μmol l^−1^, *P*<0.05, Albumin 16.6±1.6 versus 32.2±1.8 g l^−1^, *P*<0.05, Tukey's multiple comparison test). Mice reached end-stage renal failure between 7 and 10 months of age. *Lama5* has previously been shown to be critical in organ development^14^ and is associated with polycystic kidney disease^15^, but this is the first report of its involvement in a chronic renal phenotype. Recent sequencing data has identified *Lama5* mutations in focal segmental glomerular sclerosis patients^16^. Thus, this novel mutant provides supporting evidence for a role for LAMA5 in nephrotic syndromes and will enable the investigation of the pathogenic processes involved.

Our studies also revealed a mutation in the aggrecan (*Acan)* gene in pedigree MPC-227, causing an A1946V substitution in the C-type-lectin domain, which results in late-onset joint deterioration and obesity, both novel phenotypic associations with this locus (Supplementary Fig. 2). By 12 months of age mutant mice have an altered body composition with a significantly higher percentage fat mass (Supplementary Fig. 2a). In contrast to the late-onset obesity, throughout life homozygous Acan^A1946V^ mice have significantly lower absolute lean mass (Supplementary Fig. 2b). So, while metabolically obese, mutant mice are not heavier overall than littermates (Supplementary Fig. 2c). At 18 months of age mutant mice exhibit on X-ray bony deposits, particularly in the knee joints (Supplementary Fig. 2d,e). Existing mouse mutants^17^,^18^ have not recapitulated the early-onset osteoarthritis observed in patients with mutations in this gene^19^,^20^. In addition to the quantitative differences in fat mass, histopathology revealed qualitative differences in fat tissues. Adipocytes from white adipose tissue in *Acan*^*A1946V*^ mice are enlarged and there is evidence of inflammatory cell inflammation (Supplementary Fig. 2f,g). Conversely, the brown adipose tissue shows reduced accumulation of fat in the mutant mice (Supplementary Fig. 2h,i). Aggrecan expression has been identified in several cell types within adipose tissue, including preadipocytes and pericytes, and can influence adipogenesis^21^.

### *Slc4a10* is a novel late-onset hearing loss gene

We have uncovered several novel genes associated with progressive and/or late-onset hearing loss, including solute carrier family 4, sodium bicarbonate transporter, member 10 (*Slc4a10*), *Wars2*, protein tyrosine phosphatase, receptor type, Q (*Ptprq*) and zinc finger, FYVE domain containing 26 (*Zfyve26*). Here we elaborate the phenotype and characterization of the *Slc4a10* late-onset hearing loss mutant, which exemplifies the novel insights into gene function and molecular mechanisms associated with late-onset disease. Auditory phenotyping of pedigree MPC-96 at 3, 6, 9 and 12 months of age found all mice displayed a normal response to a clickbox stimulus. However, when assessed using auditory-evoked brainstem response (ABR) testing six mice were found to have mildly elevated hearing thresholds at 9 months of age thus indicating an impaired hearing function (Supplementary Fig. 3a). Subsequent screening of this pedigree at 12 months of age found these six mice had a further increase in their hearing thresholds suggesting a progressive phenotype (Supplementary Fig. 3b). After further breeding no hearing impaired mice were observed in the backcrossed G_4_ litters. However in the inter-crossed G_5_ litters mice with reduced hearing were identified, indicating a recessive inheritance.

A genome scan of G_3_ mice showed linkage to a ∼63 Mb region on chromosome 2 containing 936 genes. Subsequent mapping narrowed the critical interval to ∼12.5 Mb (Supplementary Fig. 4a). Analysis of the WGS data identified only a single high-confidence non-synonymous coding change within the mapped interval, consisting of a T-to-C transition at nucleotide 1940 of the *Slc4a10* gene (Ensembl transcript ID ENSMUST00000112480) causing a leucine-to-proline substitution at residue 647 (Supplementary Fig. 4b) A list of noncoding mutations in the minimal mapping region identified through WGS is shown in Supplementary Table 3. The presence of this lesion was confirmed using Sanger sequencing (Supplementary Fig. 4b). Only mice showing late-onset hearing impairment were homozygous for the L647P ENU-induced mutation. The L647 residue is within the transmembrane helix-containing domain of the protein (Supplementary Fig. 4c) and is conserved across species (Supplementary Fig. 4d). This mutation was predicted to be deleterious: SIFT, affect protein function (0.00); PolyPhen-2, probably damaging (0.986); and Mutation Taster, disease causing (0.999) (refs 22, 23, 24). This mouse line was named *trombone* (*trmb*).

**Figure 3:**
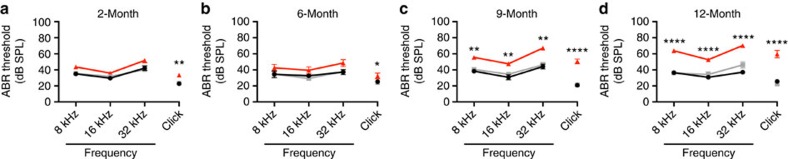
ABR phenotyping of *trombone* mice from 2 to 12 months of age. Minimum auditory detection thresholds (decibel SPL (dB SPL)) were determined using ABR and scored independently by two operators. (**a**) At 2 months of age, *Slc4a10*^*trmb/trmb*^ (homozygous) mice (*n*=44) show only a very mild elevation in ABR thresholds in comparison with *Slc4a10*^*+/trmb*^ (heterozygous) (*n*=54) and *Slc4a10*^*+/+*^ (wild-type) (*n*=18) littermates. (**b**) At 6 months of age, the very mild elevation of ABR thresholds is still observed in *Slc4a10*^*trmb/trmb*^ mice (*n*=10) compared with *Slc4a10*^*+/trmb*^ (*n*=21) and *Slc4a10*^*+/+*^ (*n*=13) littermates. (**c**) By 9 months of age, *Slc4a10*^*trmb/trmb*^ mice (*n*=26) show significantly elevated ABR thresholds (∼20 dB shift) across all frequencies tested compared with *Slc4a10*^*+/trmb*^ (*n*=25) and *Slc4a10*^*+/+*^ (*n*=6) littermates. (**d**) At 12 months of age, *Slc4a10*^*trmb/trmb*^ mice (*n*=17) continue to have elevated thresholds compared with the normal hearing *Slc4a10*^*+/trmb*^ (*n*=13) and *Slc4a10*^*+/+*^ (*n*=7) control mice. In addition, the average hearing thresholds of *Slc4a10*^*trmb/trmb*^ mice increase between 9 and 12 months of age. The *Slc4a10*^*+/trmb*^ and *Slc4a10*^*+/+*^ control mice do not show any auditory decline during the first 12 months of life, nor do they show any significant differences between their auditory thresholds at any frequency or age tested. Black circle/line, *Slc4a10*^*+/+*^; grey square/line, *Slc4a10*^*+/trmb*^; red triangle/line, *Slc4a10*^*trmb/trmb*^. Data shown are mean ±s.e.m. and significance determined using a one-way ANOVA with Tukey's multiple comparisons test comparing *Slc4a10*^*trmb/trmb*^ threshold data with the corresponding *Slc4a10*^*+/+*^ control data: *P<0.05, **P<0.01, ***P<0.001, ****P<0.0001.

To further investigate the auditory phenotype in the *trombone* model, additional mice were bred and assessed by clickbox and ABR at 2, 6, 9 and 12 months of age. All genotypes (*Slc4a10*^*+/+*^, *Slc4a10*^*+/trmb*^, and *Slc4a10*^*trmb/trmb*^) displayed a normal Preyer reflex in response to a clickbox stimulus at all ages tested. In addition, *Slc4a10*^*+/+*^ and *Slc4a10*^*+/trmb*^ mice have ABR thresholds within the normal range at all ages tested. However, while *Slc4a10*^*trmb/trmb*^ mice have similar ABR thresholds to their *Slc4a10*^*+/+*^ and *Slc4a10*^*+/trmb*^ controls at 2 and 6 months of age, by 9 months they have mildly elevated ABR thresholds at all tested frequencies (Fig. 3). At 12 months of age the auditory thresholds of *Slc4a10*^*trmb/trmb*^ mice are further elevated, displaying an increase of ⩾10 dB sound pressure level (SPL) at 8, 16 and 32 kHz, indicating a progressive late-onset auditory impairment (Fig. 3). In addition, no overt vestibular dysfunction (for example, circling, head bob, abnormal swim), craniofacial dysmorphology or weight phenotype were observed.

**Figure 4:**
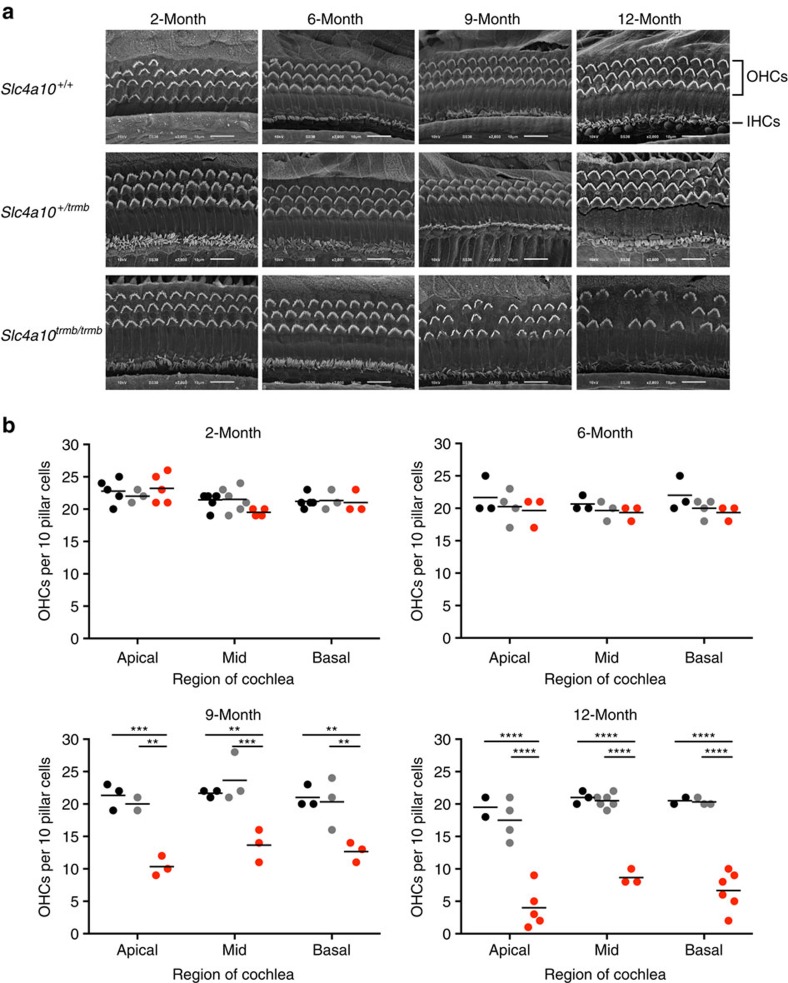
Ultrastructural analyses of the cochlear sensory epithelium reveals progressive loss of hair cell bundles in *trombone* mutant mice. (**a**) Scanning Electron Micrographs of the mid-basal coil of the cochlear sensory epithelium from *Slc4a10*^*+/+*^, *Slc4a10*^*+/trmb*^, *Slc4a10*^*trmb/trmb*^ mice at 2, 6, 9 and 12 months of age. At 2 and 6 months of age, the number and appearance of the inner and outer hair cell stereocilia bundles are is as expected and similar across all three genotypes. At 9 months, there is some loss of outer hair cell bundles in the *Slc4a10*^*trmb/trmb*^ mutant mice, which is not observed in the *Slc4a10*^*+/+*^ and *Slc4a10*^*+/trmb*^ control mice. By 12 months of age, there is a substantial loss of outer hair cell bundles in the *Slc4a10*^*trmb/trmb*^ mutant mice, which is not observed in the *Slc4a10*^*+/+*^ and *Slc4a10*^*+/trmb*^ control mice. Magnification × 2,500. Scale bars, 10 μm (**b**) Outer hair cell bundle counts in the apical, mid and basal turns of the cochlear spiral in *trombone* mice. At 2 months of age *Slc4a10*^*+/+*^ (apex *n*=5, mid *n*=7, base *n*=5), *Slc4a10*^*+/trmb*^ (apex *n*=3, mid *n*=6, base *n*=3), *Slc4a10*^*trmb/trmb*^ (apex *n*=5, mid *n*=4, base *n*=3) mice all have similar numbers of OHC bundles. At 6 months of age *Slc4a10*^*+/+*^ (apex *n*=3, mid *n*=3, base *n*=3), *Slc4a10*^*+/trmb*^ (apex *n*=4, mid *n*=3, base *n*=4), *Slc4a10*^*trmb/trmb*^ (apex *n*=3, mid *n*=3, base *n*=3) mice all still have similar numbers of OHC bundles. However, by 9 months of age *Slc4a10*^*trmb/trmb*^ (apex *n*=3, mid *n*=3, base *n*=3) mice have a reduced number of OHC bundles in all cochlear regions compared with *Slc4a10*^*+/+*^ (apex *n*=3, mid *n*=3, base *n*=3), *Slc4a10*^*+/trmb*^ (apex *n*=2, mid *n*=3, base *n*=3) mice. At 12 months of age *Slc4a10*^*trmb/trmb*^ (apex *n*=5, mid *n*=3, base *n*=6) mice show a further loss of OHC bundles in all cochlear regions compared with *Slc4a10*^*+/+*^ (apex *n*=2, mid *n*=3, base *n*=2), *Slc4a10*^*+/trmb*^ (apex *n*=4, mid *n*=6, base *n*=3) mice. Although *Slc4a10*^*trmb/trmb*^ mice show a progressive loss of OHC bundles throughout the cochlear spiral, no significant OHC bundle loss is observed in the *Slc4a10*^*+/+*^ or *Slc4a10*^*+/trmb*^ mice up to 12 months of age. *Slc4a10*^*+/+*^ (black bars), *Slc4a10*^*+/trmb*^ (grey bars), *Slc4a10*^*trmb/trmb*^ (red bars). Data shown are mean ±s.e.m. and significance determined using a two-way ANOVA with Tukey's multiple comparisons test: **P<0.01, ***P<0.001, ****P<0.0001.

To assess the ultrastructure of the cochlear sensory epithelium scanning electron microscopy was undertaken (Fig. 4). *Slc4a10*^*+/+*^ and *Slc4a10*^*+/trmb*^ mice display the expected complement of inner hair cells and outer hair cells (OHCs) up to 12 months of age, the latest age tested. At 2 and 6 months of age *Slc4a10*^*trmb/trmb*^ mice also display the expected number of inner hair cells and OHCs, with no differences in shape or organization of their stereocilia bundles (Fig. 4a). However, by 9 months of age OHC bundle loss is evident in *Slc4a10*^*trmb/trmb*^ mutant mice, and this loss progresses such that by 12 months of age <50% of OHC bundles remain (Fig. 4a). To quantify the loss, counts were made in the apical, mid and basal turns of the cochlea (Fig. 4b). This shows that in *Slc4a10*^*trmb/trmb*^ mice OHC bundle loss is occurring along the length of the cochlear spiral. There is no evidence of OHC bundle loss in *Slc4a10*^*+/+*^ and *Slc4a10*^*+/trmb*^ mice up to 12 months of age.

**Figure 5:**
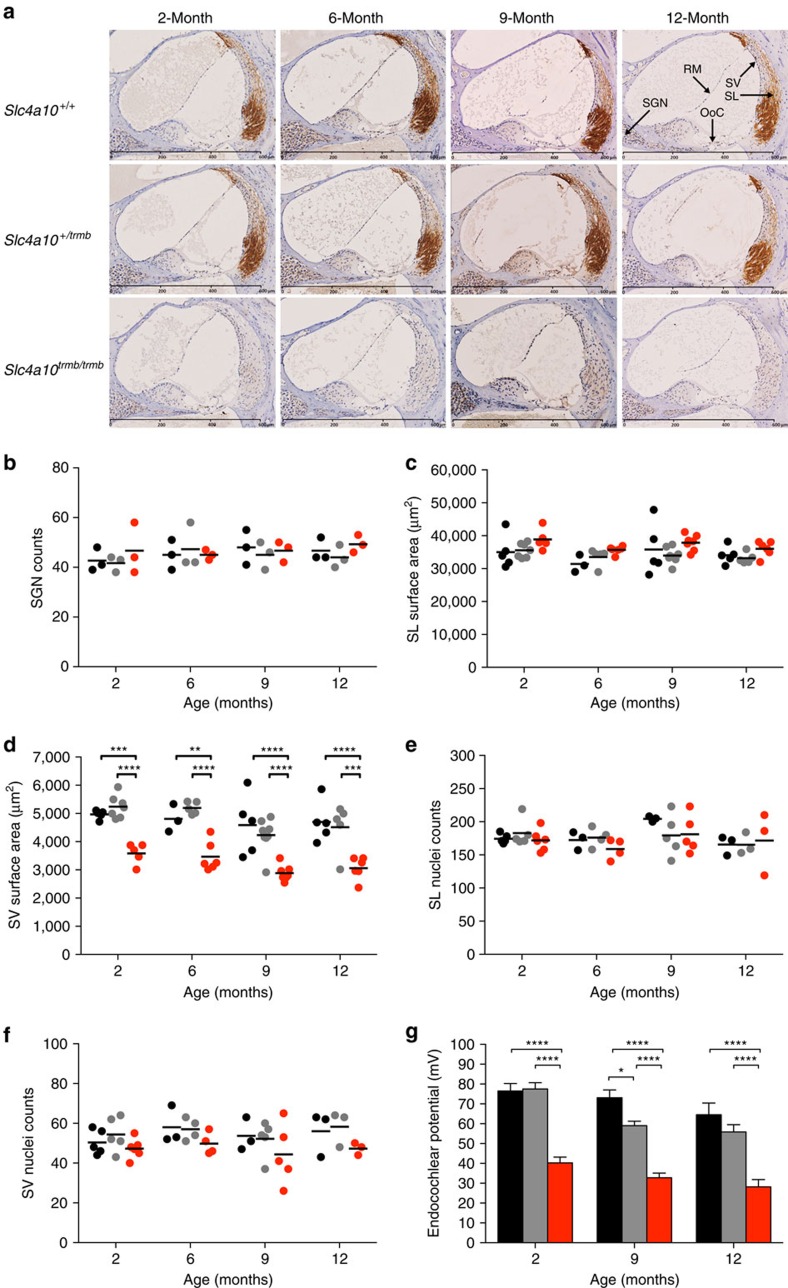
Cochlear expression of Slc4a10 morphology of the lateral wall and endocochlear potential in *trombone* mice. (**a**) Immunohistochemical DAB staining of cochlear sections identifies Slc4a10 expression in the SL fibrocytes of *Slc4a10*^*+/+*^ and *Slc4a10*^*+/trmb*^ mice. No DAB staining of *Slc4a10*^*trmb/trmb*^ sections is observed at any of the time points tested. Scale bar, 600 μm. Number of cochleae imaged (one per mouse) for each genotype at 2-, 6-, 9- and 12 months of age were: *Slc4a10*^*+/+*^ *n*=5, 5, 6, 6; *Slc4a10*^*+/trmb*^ *n*=7, 4, 5, 6; and *Slc4a10*^*trmb/trmb*^ *n*=4, 3, 5, 6, respectively. (**b**) SGN counts in *Slc4a10*^*+/+*^, *Slc4a10*^*+/trmb*^ and *Slc4a10*^*trmb/trmb*^ mice shows there are no differences across genotype, or with age. Counts averaged from individual mice (*N*=3) for each genotype at each age. (**c**) Analysis of the cross-sectional surface area of the SL shows there are no differences across genotypes, or with age. (**d**) Analysis of the SV show there is a significant reduction in the cross-sectional surface area in *Slc4a10*^*trmb/trmb*^ mice compared with *Slc4a10*^*+/+*^ and *Slc4a10*^*+/trmb*^ mice, and this is consistent across the ages tested. The SL and SV surface areas are averaged from individual mice for each genotype at 2-, 6-, 9- and 12 months of age: *Slc4a10*^*+/+*^ *n*=5, 3, 5, 5; *Slc4a10*^*+/trmb*^ *n*=7, 5, 8, 5; and *Slc4a10*^*trmb/trmb*^ *n*=5, 6, 7, 6, respectively. (**e**,**f**) Comparison of SL and SV nuclei counts shows there are no differences across genotype, or with age. The nuclei counts are averaged from individual mice for each genotype at 2-, 6-, 9- and 12 months of age: *Slc4a10*^*+/+*^ *n*=5, 3, 3, 3; *Slc4a10*^*+/trmb*^ *n*=5, 4, 4, 3; and *Slc4a10*^*trmb/trmb*^ *n*=6, 5, 4, 3, respectively. (**g**) The endocochlear potential is chronically reduced in *Slc4a10*^*trmb/trmb*^ mice compared with controls. Measurements averaged for each genotype at 2-, 9- and 12 months of age: *Slc4a10*^*+/+*^ *n*=6, 6, 8; *Slc4a10*^*+/trmb*^ *n*=10, 12, 11; and *Slc4a10*^*trmb/trmb*^ *n*=8, 6, 9, respectively. *Slc4a10*^*+/+*^ (black circles/bars), *Slc4a10*^*+/trmb*^ (grey circles/bars), *Slc4a10*^*trmb/trmb*^ (red circles/bars). Data correspond to mean±s.e.m., and statistical significance determined using two-way ANOVA with Tukey's multiple comparisons test. *P<0.05; **P<0.01; ***P<0.001, ****P<0.0001. ANOVA, analysis of variance.

To assess the expression of *Slc4a10* within the cochlea, mid-modiolar histological sections were prepared and immunostained using an anti-Slc4a10 antibody. For *Slc4a10*^*+/+*^ and *Slc4a10*^*+/trmb*^ cochlear sections, immunohistochemical staining was observed in the spiral ligament (SL) fibrocytes throughout all cochlear turns at 2, 6, 9 and 12 months of age (Fig. 5a). The strongest staining was detected in the type II fibrocytes beneath the spiral prominence and the type V fibrocytes in the suprastrial region. There was no staining of additional cochlear structures, for example, organ of Corti (OoC), Reissner's membrane (RM), or spiral ganglion neurons (SGN) (Fig. 5a). For *Slc4a10*^*trmb/trmb*^ cochlear sections no staining of the SL was detected at any of the time points tested, implying a loss-of-function mutation (Fig. 5a). In addition, assessment of the cochlear sections identified a similar number of SGNs present across all genotypes at each time point investigated (2, 6, 9 and 12 months of age) (Fig. 5b).

To assess the consequence of the *trombone* mutation on the structure of the cochlear lateral wall morphometric analyses were performed. Utilizing hematoxylin and eosin stained mid-modiolar cochlear sections the cross-sectional surface area of the SL, and the closely apposed stria vascularis (SV), were measured in the mid-basal cochlear turn. This identified that the cross-sectional surface area of the SL was consistent across all genotypes, at all ages (Fig. 5c). Assessment of the SV identified that the cross-sectional surface area was similar between *Slc4a10*^*+/+*^ and *Slc4a10*^*+/trmb*^ mice at each time point. However, the cross-sectional surface area of the SV was reduced by >20% in *Slc4a10*^*trmb/trmb*^ mice (Fig. 5d). Interestingly, SL and SV nuclei counts demonstrate there is not a statistically significant difference in the total number of cells within either of these structures across genotype (*Slc4a10*^*+/+*^, *Slc4a10*^*+/trmb*^ and *Slc4a10*^*trmb/trmb*^) or age (2, 6, 9 and 12 months) (Fig. 5e,f).

The SV is critical for generating the extracellular fluid (endolymph) found in the scala media, which bathes the apical surface of the auditory sensory cells. By pumping K^+^ into the scala media the SV generates a high K^+^ concentration and large electrical potential, known as the endocochlear potential (EP), in this extracellular space compared with that of the perilymph-containing scala tympani. The EP is essential for the process of auditory transduction, establishing an electrochemical gradient that drives cations from the endolymph into the sensory cells via mechanically gated channels to cause depolarization of, and subsequent neurotransmitter release from, the sensory cells in a process known as mechanoelectrical transduction. To assess if SV function is compromised, the EP was measured in *trombone* mice at 2, 9 and 12 months of age. At 2 months of age the averaged EP values for *Slc4a10*^*+/+*^ and *Slc4a10*^*+/trmb*^ mice were similar at 76 mV (range 62–89 mV) and 77 mV (range 53–89 mV), respectively. However, the averaged EP value for age-matched *Slc4a10*^*trmb/trmb*^ mice was significantly lower at 40 mV (range 29–55 mV) (Fig. 5g). At 9 months of age the averaged EP values for *Slc4a10*^*+/+*^ and *Slc4a10*^*+/trmb*^ mice were 73 mV (range 57–82 mV) and 59 mV (range 40–69 mV), respectively. Again, the averaged EP value for age-matched *Slc4a10*^*trmb/trmb*^ mice was significantly lower at 32 mV (range 27–41 mV) (Fig. 5g). At 12 months of age, the averaged EP values for *Slc4a10*^*+/+*^ and *Slc4a10*^*+/trmb*^ mice were 65 mV (range 42–82 mV) and 55 mV (range 44–81 mV), respectively; whereas the averaged EP value for age-matched *Slc4a10*^*trmb/trmb*^ mice was significantly lower at 28 mV (range 15–46 mV) (Fig. 5g). The observed age-related decline in EP is only significant for the *Slc4a10*^*+/trmb*^ mice (*P<*0.0001).

Recent studies of an *Slc4a10* targeted KO mouse mutant (*Slc4a10*^−*/*−^) have identified a role for *Slc4a10* in maintaining intracellular chloride and bicarbonate concentration in retinal neurons, demonstrating that loss of *Slc4a10* in the retina leads to impaired visual function in the *Slc4a10*^*−/−*^ KO mouse^25^. To ascertain if the *Slc4a10*^*trmb/trmb*^ mice also display a retinal phenotype electroretinography (ERG) was undertaken. To enable this, the *trombone* allele was rederived on, and backcrossed to, C57BL/6J, a strain suitable for ERG studies. The ERG analysis showed that *trombone* mice display a very similar, albeit milder, retinal phenotype to the *Slc4a10*^−*/*−^ KO mice (Supplementary Fig. 5a). Overall, compared with wild-type littermate mice, *Slc4a10*^*trmb/trmb*^ mice showed: grossly similar dark-adapted irradiance-response curves for a- and b-waves (Supplementary Fig. 5b,c) significant differences in phase/timing of flicker frequency responses (delayed in mutant) especially in 7–15 Hz responses (Supplementary Fig. 5d); and, smaller and slower light-adapted responses showing significant differences in amplitude and implicit time at higher flash intensities (Supplementary Fig. 5e–i). The similar phenotype supports our hypothesis that the *trombone* allele causes loss-of-function. In summary, the *trombone* mutant (*Slc4a10*^*L647P*^) underlines the utility of characterising mutations with late-onset phenotypes, uncovering novel genes and insights into the underlying molecular mechanisms. Age-related hearing loss in *trombone* mice is preceded by changes to the structure of the SV and by defects in endocochlear potential.

## Discussion

We have established a large-scale ENU G_3_ recessive screen for age-related disease phenotypes. The aim has been to provide a wider understanding of the genetic pathways that contribute to disease in an ageing context. Overall, we have demonstrated that ENU phenotype-driven screens have the capacity to uncover a significant number of phenotypes that display late-onset disease and which are caused by mutations in novel genes, hitherto not associated with known functions in age-related disease. In total we have examined over 150 G_3_ pedigrees, identifying 105 distinct mutant lines, including 27 lines with late-onset phenotypes.

A significant number of pedigrees revealed multiple phenotypes in our screening pipeline. This is not surprising given the large size of the pedigrees and the relatively comprehensive phenotype screens that we have undertaken. However, most importantly, we find that around one quarter of all mutant phenotypes are detectable from 7 months onwards, indicating that a significant number of mutations leading to alterations in gene function are only manifest in an ageing context. This high hit rate indicates that the ongoing analysis of further pedigrees will be a productive route for the recovery of considerable numbers of additional age-related disease loci.

Employing a high-level ontological analysis, it is clear that we have identified late-onset phenotypes, which span a wide disease spectrum. We would expect that the random nature of ENU mutagenesis provides an unbiased route to the identification of late-onset disease loci. Our data support the conclusion that this approach represents a powerful approach for revealing late-onset phenotypes in any disease domain, as much as it has already been shown to be a productive approach for the identification of early-onset phenotypes^3^,^4^,^6^.

We have employed WGS to identify the mutations underlying individual phenotypes. This approach revealed the sequence alteration in 44 out of 47 mutant phenotypes investigated. The 44 mutations identified comprised missense, splice site, nonsense mutations and an intracisternal A-particle insertion, in each case likely to compromise gene function. A wide range of ENU mutagenesis programmes have reported a very high frequency of coding changes, particularly missense, that underlie mutant phenotypes and our results are in agreement with these findings. For three mutant phenotypes investigated there are no coding or splice-site mutations that could account for the observed phenotype. It is unclear whether or not the functional change is located in noncoding sequences, but the availability of whole-genome sequence allows us to document all the sequence variants in the mapped region and potentially to test individual variants.

Out of 27 late-onset phenotypes we have so far identified the underlying lesion in 12, and additional mutations remain to be confirmed. We report in Table 3 the novel gene functionality that has been revealed. These results indicate the power of phenotype-driven ENU mutagenesis screens, which make no *a priori* assumptions about gene function or the pathways involved in disease, to uncover novelty, in this case the underlying basis of age-related disease. In several cases, the identification of novel genes and pathways are likely to open up new lines of enquiry into the physiological and pathological bases of late-onset phenotypes.

As an example of the novel gene function associated with ageing phenotypes, we report the characterization of the *trombone* late-onset hearing loss mutation. The *trombone* mice display a late-onset progressive hearing loss, which is due to a loss-of-function mutation in the sodium-coupled bicarbonate transporter (NCBT), *Slc4a10*. Before this investigation, *Slc4a10* has not been reported to be associated with auditory dysfunction in the mouse or in human studies. However, a study has shown that *Slc4a10* KO mice have smaller brain ventricles and an increased threshold to experimentally induced seizures compared with wild-type animals. They also report that *Slc4a10* is important for regulating neuronal intracellular pH and excitability, and suggest that because *Slc4a10* is involved in solute transport within the epithelial cells of the choroid plexus it is likely to contribute to secretion of cerebrospinal fluid^26^. Interestingly, *Slc4a7*, another NCBT closely related to *Slc4a10*, has been linked to auditory dysfunction and retinal impairment. *Slc4a7* also acts to maintain intracellular pH playing a vital role in the efficient disposal of acid (H^+^) generated by neuronal and sensory receptor activity, which is an essential requisite of sensory transduction. Mice lacking *Slc4a7* develop auditory impairment and blindness due to degeneration of sensory receptors in the inner ear and eye, respectively^27^. Auditory impairment in these mice is present from one month of age, and is concomitant with loss of hair cells and morphological changes in the SV and SL. In addition to the very similar phenotype, the expression pattern of *Slc4a7* is the same as *Slc4a10*, with expression in the type II and type V cochlear fibrocytes. It was hypothesized that impaired ion transport by the SL fibrocytes in *Slc4a7* KO mice may lead to hair cell degeneration and consequently auditory impairment.

Currently very little is known regarding the pathological changes occurring within the human auditory system that result in age-related hearing loss, also known as presbycusis. However, seminal work by Schuknecht has led to several pathological subtypes of presbycusis to be proposed; these include: ‘sensory', involving hair cell loss; ‘strial', involving degeneration of the SV and reduction in EP; and ‘neural', involving loss of SGNs^28^,^29^,^30^. The hearing impairment observed in *trombone* mice is concomitant with a progressive loss of OHCs, but the underlying mechanism likely involves a chronically reduced EP that precedes OHC loss and hearing impairment. As such, our findings suggest that *trombone* is likely a model of *strial* presbycusis, and that *Slc4a10* is a candidate gene for human age-related hearing loss. Furthermore, *trombone* will serve as a model to learn more about the functional requirement of bicarbonate transporters in the lateral wall and as a model to test the efficacy of gene-delivery techniques to target the cochlear lateral wall and ameliorate hearing loss.

Ageing screens as described here have the potential to provide models that will underpin improved preclinical studies of therapeutic interventions, particularly as the disease occurs in the context of an ageing physiology. Many mouse models currently used to develop therapies for age-related and chronic diseases have early-onset phenotypes that are often acute. As a consequence, potential therapies are assessed against the background of a rapid and aggressive disease. Moreover, pharmacodynamics may change with ageing; for example immunosenescence will influence the effect of immunization in the aged^31^, while mitochondrial dysfunction can result in adverse drug reactions^32^. Thus the models of age-related disease we are identifying will not only aid our understanding of disease pathogenesis but also provide more refined preclinical models with a greater window of opportunity for therapeutic intervention.

In conclusion, we have established a large-scale phenotype-driven screen for genes associated with age-related disease and successfully identified mutants resulting in such pathologies. These novel mutants will not only aid our understanding of pathogenic processes occurring during ageing but act as robust preclinical models of disease.

## Methods

### Mice

All animals were housed and maintained in the Mary Lyon Centre at MRC Harwell, under specific pathogen-free conditions in individually ventilated cages, with environmental conditions as outlined in the Home Office Code of Practice. Home Office ethical approval was granted under project licence 30/3070 and mice were killed by Home Office Schedule 1 methods. ERG experiments were performed at the University of Oxford in compliance with the ARVO statement for the Use of Animals in Ophthalmic and Vision Research and with relevant institutional and UK Home Office approval. All screened mice used in this study are G_3_ animals with a mixed C3H.Pde6b+ and C57BL/6J genetic background. Further investigation of the *trombone* auditory phenotype was undertaken on G_4_ to G_7_ mice backcrossed to C3H.Pde6b+. To enable ERG phenotyping of *trombone* mice, the line was rederived by IVF using sperm from the G_1_ male and backcrossed to C57BL/6J for two generations before inter-crossing.

### Generation of mutagenized pedigrees

The ENU mutagenesis protocol has been described previously^4^. The Harwell Ageing Screen is a large-scale ENU mutagenesis screen to generate mouse models of age-related disease. G_3_ recessive pedigrees, of ∼100 mice, are bred and enter a phenotyping pipeline comprising recurrent assessment (to 18 months of age) across a wide range of disease areas. Briefly, C57BL/6J male mice (G_0_) were treated with ENU doses of 1 × 120 mg kg^−1^, and then 2 × 100 mg kg^−1^ with a week between each dose. The mice were then bred with wild-type ‘sighted C3H' (C3H.Pde6b+) females^11^. The resulting G_1_ males were bred with wild-type C3H.Pde6b+ females to produce G_2_ females, which were genotyped for the hypomorphic *Cdh23* allele, *ahl1*^12^,^33^. G_2_ females that were wild-type for *Cdh23* were backcrossed to their G_1_ fathers to generate G_3_ offspring. Some phenotypes, such as glucose tolerance, are strongly modified by sex and circadian screening necessitated individually housing mice for up to 3 weeks, thereby limiting the screen to female mice because of the potential problems trying to rehome males together after being separated or housing males individually for extended periods Therefore some screens utilised a single sex for the initial screening but upon identification the other sex was screened as well. The G_3_ mating scheme results on average in one in eight G_3_ mice homozygous for any particular ENU-induced mutation. Binomial probability calculations indicate that a cohort of 50 single sex mice will yield four homozygotes with a probability of ∼85% and five with a probability of ∼75%. Thus we aim for a pedigree size of around 100 to enter the ageing screen.

### Phenotyping platforms

An overview of the phenotyping timetable is presented in Table 1 and is also summarized in Supplementary Fig. 1b. Additional time points may be added for specific phenotypes of interest. Furthermore, additional phenotypic investigations of individual pedigrees may be undertaken that are unsuitable for high-throughput application within the core pipeline. These include gait analysis (Cleversys or Locotronic), metabolic caging, Comprehensive Lab Animal Monitoring System analysis, optocoherence tomography, as well as behavioural tests (elevated plus maze, Morris water maze, startle/pre-pulse inhibition). For some tests a single sex was analysed initially, primarily for logistical reasons, but if an abnormal phenotype was suspected then the remaining mice were screened as well.

All primary phenotyping of ENU mutagenized pedigrees is performed blinded as genotypes are not determined until after a phenotype is identified and the majority of phenotyping data is analysed *post hoc*. For downstream analysis experimenters are blinded to the genotype and mice are housed in cages containing wild-type, heterozygous and homozygous mutants bred from heterozygous inter-cross matings, and randomly assigned at weaning before genotyping of individuals.

### Linkage analysis and DNA sequencing

DNA from affected mice and littermates was prepared from ear biopsies and used for linkage mapping utilizing the Illumina GoldenGate Mouse Medium Density Linkage Panel (Gen-Probe Life Sciences Ltd, UK). DNA from G_1_ or affected G_3_ mice was prepared for either WGS using the Nucleon BACC2 Genomic DNA Extraction System (GE Healthcare Life Sciences), a library generated, and a single lane of paired-end sequencing (100 nt) undertaken employing the Illumina HiSeq platform (Oxford Genomics Centre, Wellcome Trust Centre for Human Genetics). The paired-end Illumina reads, 100 nt in length for each G_1_ was aligned to the reference genome (NCBIM38/mm10) using Burrows-Wheeler Aligner^34^. Single-nucleotide variants (SNVs) in each alignment were detected using the unified GenotypeCaller in the Genome Analysis Toolkit (GATK)^38^ with mouse dbSNP version 137 as the background SNP set and default parameters. SNV sites that obtained a variant quality score <100 (this is a Phred scaled quality score, −10 × log (1−p), where p is the probability of the SNV being called incorrectly), or had a read depth <3 were removed from any further analysis. The remaining SNVs, termed high-confidence mutations, were then compared with the precompiled SNPs found in 17 inbred strains from the Mouse Genome Project^35^ and an in-house library of SNVs. The overlapping sites were removed resulting in a final list of unique ENU SNVs for each G_1_. These SNVs were then functionally annotated (for example, missense, intronic and so on) using NGS-SNP^36^. All high-confidence mutations are available on MouseBook. For individual mutant lines SNVs were confirmed by Sanger sequencing of affected mice.

### Data import

Phenotype data was initially captured in different formats including an in-house LIM system (Anonymus), Excel spreadsheets, structured files (for example, CSVs) or plain text. For automatic phenotype detection, customised code was generated for each data type, which imported the data into a common data format. This data was transferred to a single import engine that validated and then added the data to the Ageing database as appropriate. Raw data validation was carried out using the standard operating procedure in IMPRESS and Anonymus. The former was used to validate phenotype parameter features such as unit type and the minimum, maximum value whilst the latter was used to validate mouse features, for example, mouse id, sex, date of birth.

### Outlier detection and visualization

To automatically detect potential phenodeviants we developed approaches to identify animals with abnormal data values from a genetically mixed data set. Where there is no baseline phenotype data to identify potential outliers inferential statistical tests are not applicable. We therefore first constructed a reference range from the whole Ageing pipeline data set, as commonly used for clinical chemistry data^37^. For each phenotype parameter we employed all data values and constructed a reference range by establishing the percentile positions for the entire data set, providing high and low critical values. Parameter values above the high critical value, or below the low critical value, are marked as outliers.

Phenodeviancy calls were made if three or more animals produce a value either higher or lower (in the same direction) than the calculated reference range. The range used, 95.8%, approximately equates to ±2 s.d. The exception is ABR (auditory brainstem response), where two or more animals were used to make a phenodeviancy call. This approach generated a series of phenodeviancy calls for each measured time point. When phenodeviancy occured at >50% of the subsequent time points from the time of onset (that is, it is not a random temporal outlier) we considered this a positive phenotype call within the pedigree. Phenodeviancy calls were annotated with the appropriate Mammalian Phenotype term defined in IMPReSS. Procedure values and phenodeviancy calls are exported to MouseBook where bespoke graphs and advanced searches for every pedigree, procedure and parameter combination are displayed.

### Determination of false discovery rate

To assess the false discovery rate, a reference range was created using purely IMPC C57BL/6NTac wild-type data, at a single time point, using comparable phenotype procedures that are used in the ageing screen. This produced a set of reference ranges for the 10 matching procedures and 90 comparable parameters. We then created synthetic pedigrees by selecting mice at random from IMPC wild-type animals. The pedigree size was determined by randomly selecting an ageing pedigree, and then employing the number of animals in the selected pedigree as the size of the IMPC synthetic pedigree. These pedigree sizes have a mean size of 92 animals and a standard deviation of 46 (from 157 total pedigrees within the ageing data set). Outliers were then identified for each random sample, using the previously calculated reference ranges for each parameter. The number of simulated pedigrees with ⩾3 outliers (either high or low) was then recorded. This simulation was then repeated 100,000 × to provide an estimation of the FPR for each parameter (number of outlier calls/total number of samples), and the total number of calls for each phenotype procedure.

### Auditory-evoked brainstem response

Mice were anaesthetized with ketamine (10%v/v) and xylasine (5% v/v) administered at the rate of 0.1 ml/10 g body mass. Animals were placed on a heated mat inside a sound attenuated chamber. Electrodes (Grass Telefactor F-E2-12) were placed subdermally over the vertex (active), right mastoid (reference), and left mastoid (ground). ABR responses were collected, amplified and averaged using TDT System III (Tucker Davies Technology, Alachua, FL, USA) in conjunction with SigCal and SigGen (Biosig) software. Click stimuli consisted of a 0.1 ms broadband click of alternating polarity. Tone-burst stimuli totalled 7-ms duration, including 1 ms rise/fall time; frequencies used were 8, 16 and 32 kHz. Recordings were performed beginning at 95 dB SPL and decreased in 5 dB increments. ABR thresholds were defined as the lowest dB SPL level at which an ABR trace pattern could be clearly distinguished. Animals were recovered using anaesthetic reversal agent Antipamezole (Antisedan, 0.1 ml 1% v/v). For the analysis of the *trombone* model the number of mice tested for each genotype at 2-, 6-, 9- and 12 months of age were: *Slc4a10*^*+/+*^ *n*=18, 13, 6, 7; *Slc4a10*^*+/trmb*^ *n*=54, 21, 25, 13; and *Slc4a10*^*trmb/trmb*^ *n*=44, 10, 26, 17, respectively.

### Slc4a10 genotyping

DNA was extracted from ear biopsies and used as template for PCR in the presence of the double-stranded DNA-binding dye LCGreen, and a 3′-blocked oligonucleotide probe that binds directly over the *Slc4a10* lesion matching the mutant *trombone* sequence. An asymmetric exhaustive PCR was performed using five times the amount of reverse primer (and probe) compared with forward primer. Five microlitre HotShot master mix; 1 μl LCGreen; 0.1 μl *Slc4a10*^*trmb*^ forward—5′- CTC GTC TGC TAC ATC ACC C -3′ (20 ng μl^−1^); 0.5 μl *Slc4a10*^*trmb*^ reverse—5′- CGA GTA TTG TGT CAG CAG TTC -3′ (20 ng μl^−1^); 0.5 μl *Slc4a10*^*trmb*^ forward probe—5′- GAA GTT GTT TGA GCC CAG TGA AAC CTA -3′-3NHC3 (20 ng μl^−1^); 2 μl DNA (1/10 dilution); and 0.9 μl ddH_2_0. Cycling conditions used were: 95 °C 2 min, then 55 cycles of 95 °C 30 s–60 °C 30 s–72 °C 30 s, followed by probe hybridisation 95 °C 30 s–25 °C 30 s–15 °C 30 s. Hybridised products were then heated on a LightScanner (Idaho Technology) and the fluorescence emitted by bound LCGreen was monitored using a high-resolution melting curve analysis and analysed using manufacturer's software. As the probe:PCR amplicon duplex melts the LCGreen dye is released and fluorescence decreases. The probe matches the mutant sequence and as such melts at a slightly higher temperature than the wild-type sequence, which harbours a mis-match. Using this method wild-type, heterozygous and homozygous mutant genotypes can be identified.

### Scanning electron microscopy (SEM)

Animals were killed and excised inner ears were fixed overnight in 2.5% gluteraldehyde in 0.1 M phosphate buffer (Sigma-Aldrich). Fixed ears were decalcified for 48 h in 4.3% EDTA in 0.1 M phosphate buffer (Sigma-Aldrich). Fine dissection was performed to reveal the organ of Corti, before osmium tetroxide (Agar Scientific)—thiocarbohydrazide (Fluka) (OTOTO) processing (adapted from ref. 38) was carried out. The inner ears were then dehydrated through increasing strength ethanol solutions (Fisher Scientific) and critical point dried using an Emitech K850 (EM Technologies LTD). The specimens were then mounted on stubs using silver paint (Agar Scientific) and sputter coated with platinum using a Quorum Q150T sputter coater (Quorum Technologies). Prepared cochleae were visualised with a JEOL LSM-6010 (Jeol Ltd.) scanning electron microscope. Hair cell counts were performed by counting the number of OHCs adjacent to 10 pillar cells, for the analysis the cochlea was divided into three separate regions (turns), apical (<90° from apex), mid (180–360° from apex) and mid-basal (360–540° from apex). Ears from at least three mice were analysed for each genotype at each turn and time point.

### Cochlear histology

Mice were killed, heads removed, skinned and bisected along the midline before fixation in 10% neutral buffered formalin (Surgipath). Fixed specimens were decalcified, dehydrated and embedded in paraffin wax, 5 μm sagittal sections were obtained and H&E stained using standard protocols.

### Electroretinography

ERG testing was used to examine retinal function in adult (11–12 week old) mice. *N*=5 homozygous *trombone* mutant mice (*Slc4a10*^*trmb/trmb*^) were tested, with the same number of age and sex matched homozygous wild-type littermates used as controls. Mice were housed under standard 12:12 light–dark cycle with food and water available *ad libitum*.

ERG preparation and testing were performed in a similar manner to that described previously^39^. Before testing, mice were dark-adapted overnight (>16 h) and all experimental preparation was performed under dim red illumination. Animals were anaesthetised via intraperitoneal injection of 80 mg kg^−1^ ketamine (Vetalar, Pfizer Animal Health, Sandwich, UK) and 10 mg kg^−1^ xylazine (Rompun, Bayer HealthCare, Newbury, UK) in sterile water for injection. Pupils were fully dilated with tropicamide and phenylephrine eye drops (Minims tropicamide 1% w/v and Minims phenylephrine hydrochloride 2.5% w/v, both Bausch & Lomb, Kingston-Upon-Thames, UK). Animals were placed on a heated platform, maintained at 38 °C using a circulating pump–water bath. A DTL-type silver-coated nylon thread active electrode (DTL Plus Electrode; Diagnosys LLC, Cambridge, UK) was modified to include a custom-made contact lens of clear Aclar film (Honeywell International, Inc., supplied by Agar Scientific, Stansted, UK). This was positioned concentrically on the cornea using 1% hypromellose eye drops (Isopto alkaline, Alcon Laboratories, Camberley, UK) to provide good electrical contact and to maintain corneal hydration. Active electrodes were placed on both eyes and recorded the ERG of each eye independently. Platinum needle electrodes in the forehead and at the base of the tail served as common reference and ground electrodes, respectively. All recordings were made in a custom-made light-tight Faraday cage. ERG signals were differentially amplified and digitized at a rate of 5 kHz using an Espion E2 console (Diagnosys LLC, Cambridge, UK) that also generated and controlled the light stimuli which were delivered in a Ganzfeld dome.

In dark-adapted testing, an intensity response curve was first generated for single-flash responses elicited by brief (4 ms) flashes of white light (6,500 K light-emitting diode source) on a dark background. Recording began at the dimmest stimulus intensity and was then increased across a >7 log unit range (−6 to 1.4 log cd.s m^−2^) in up to log unit steps with the following response averaging and inter-stimulus interval (ISI) times used: −6 to −5 log cd.s m^−2^; 16 responses averaged with an ISI of 3 s; −4 to −3 log cd.s m^−2^; nine responses, ISI=4 s; −2 log cd.s m^−2^; nine responses, ISI=8 s; −1 log cd.s m^−2^; four responses, ISI=16 s; 0 log cd.s m^−2^; four responses, ISI=32 s; 1 log cd.s m^−2^; four responses, ISI=64 s; 1.4 log cd.s m^−2^, single response.

After recording the dark-adapted intensity response curve, an increasing flicker series was recorded at a fixed stimulus flash intensity (−2 log cd.s m^−2^). Flicker ERG responses were recorded at the following rates (Hz): 0.5, 1, 2, 3, 5, 7, 10, 12, 15, 18, 20 and 30. Waveforms were recorded in stimulus synchronised response epochs (sweeps) of 500 ms duration and either 20 (0.5–3 Hz) or 30 (5–30 Hz) sweeps were recorded and an averaged response was generated after excluding the first sweep per step in all cases.

After dark-adapted recordings were completed, animals were exposed to a full-field 30 cd.s m^−2^ white background (6,500 K LED source, as stimulus) for at least 10 min. Light-adapted ERGs were then recorded in response to a range of white flash stimuli (−0.5 to 1.4 log cd.s m^−2^ in ∼half log unit steps, 20 responses averaged per step, ISI = 1 s in all cases) superimposed on the constant background.

For single-flash responses, the amplitude and latency of the major ERG components (a- and b-waves for dark-adapted, b-wave only for light-adapted) was measured (Espion software; Diagnosys LLC) using automated and manual methods. By convention, b-wave amplitude is measured from the a-wave trough (where present) and a-wave amplitude is measured from baseline to a-wave trough. Implicit times of both waveforms was measured from stimulus onset to the peak amplitude.

Unfiltered traces were used for dark-adapted responses whereas in light-adapted records both a band-pass filter (between 0.625 and 300 Hz) and a 50 Hz notch filter were applied.

For flicker responses the ERG waveform becomes sinusoidal at higher stimulus frequencies (ie, >5 Hz) and discreet a- and b-waves are not distinguishable. Thus the peak-to-peak amplitude of the waveform following the first stimuli of the sweep was measured instead and the delay until the first positive deflection was also quantified to estimate response timing. These records were band-pass filtered between 0.625 and 300 Hz and a 50 Hz notch filter was also applied. In all cases, values were obtained for records from both eyes of each animal and then a mean value was calculated per individual to be used in statistical analysis.

### DAB immunolabelling

Mice were euthanized, heads removed, skinned and bisected along the midline before fixation in 10% neutral buffered formalin (Surgipath). Fixed specimens were decalcified, dehydrated and embedded in paraffin wax. For immunohistochemistry, 5 μm sagittal sections were mounted on charged slides. Slides were dewaxed, incubated with 3% hydrogen peroxide, and antigen retrieval performed using 10 mM citrate buffer. Sections were then incubated with an anti-Slc4a10 antibody (1:200, sc-161917) for one hour at room temperature. Control sections were incubated with primary antibody that had been incubated with the immunizing peptide. Sections were rinsed, and incubated with a biotinylated rabbit anti-goat secondary antibody (1:400, DAKO), rinsed again, incubated with Streptavidin-peroxidase complex (VECTASTAIN elite ABC kit, Vector Laboratories), and rinsed again. A peroxide-based immunohistochemical method utilising diaminobenzidine (DAB) was used to detect staining. The DAB solution (DAKO) was added to the sections. Once the brown stain became visible slides were washed with PBS. Sections were counterstained with haematoxylin, cleared and mounted

### Endocochlear potential measurements

These were performed as previously described by Teubner *et al*^40^. Briefly, mice were intraperitonealy anaesthetised with sodium thiobutabarbital (0.1 mg g^−1^ bodyweight) (Inactin, Sigma, Deisenhofen, Germany). The cochleae were exposed by a ventral approach and access to the basal scala media gained by thinning the bone over the SL. Borosilicate capillaries filled with 0.5 M NaCl were used as glass electrodes connected to a FD223 (World Precision Instruments) differential electrometer, which was used to measure the endocochlear potential.

### Data availability

All raw data and automatically annotated phenotype data and SNVs are available via the publically accessible data portal MouseBook (http://www.mousebook.org/), and metadata can be found at http://www.mousephenotype.org/harwellageingscreen. The Whole Genome Sequencing data has been deposited to the NCBI Short Read Archive database (https://www.ncbi.nlm.nih.gov/Traces/sra/sra.cgi?) under accession number PRJNA322401. Individual SRA biosample IDs are as follows; G1-C57BL/6J-C3H.pde6+ mice; MPC-81, SAMN05172945; MPC-91, SAMN05172946; MPC-96, SAMN05172947; MPC-102, SAMN05172945; MPC-107, SAMN05177793; MPC-173, SAMN05188717; MPC-178, SAMN05188718; MPC-184, SAMN05188720; MPC-185, SAMN05188721; MPC-187, SAMN05193605; MPC-191, SAMN05193608; MPC-200, SAMN05178551; MPC-201, SAMN05178552; MPC-203, SAMN05178554; MPC-205, SAMN05178563; MPC-214, SAMN05178564; MPC-225, SAMN05178565; MPC-227, SAMN05178566; MPC-231, SAMN05178611; MPC-232, SAMN05178612; MPC-234, SAMN05179116; MPC-236, SAMN05179117; MPC-242, SAMN05179118; MPC-246, SAMN05179119; MPC-264, SAMN05208809; MPC-265, SAMN05185998; MPC-267, SAMN05185999; MPC-269, SAMN05186000; MPC-276, SAMN05186001; MPC-282, SAMN05186002; MPC-285, SAMN05186003; MPC-290, SAMN05188407; MPC-291, SAMN05188408; MPC-292, SAMN05188409, MPC-294, SAMN05188410, and for G3-C57BL/6J-C3H.pde6+ mice; MPC-125, SAMN05177794; MPC-151, SAMN05177795; MPC-169, SAMN05188716; MPC-188, SAMN05193606; MPC-190, SAMN05193607; MPC-202, SAMN05178553.

## Additional information

**How to cite this article:** Potter, P. K. *et al*. Novel gene function revealed by mouse mutagenesis screens for models of age-related disease. *Nat. Commun.* 7:12444 doi: 10.1038/ncomms12444 (2016).

## Supplementary Material

Supplementary Information Supplementary Figures 1-5 and Supplementary Tables 1-3

## Figures and Tables

**Table 1:** The Harwell Ageing screen phenotyping pipeline.

**Test**	**Phenotypic Area**	**Group**	**Age (weeks)**
ECG	Cardiac	All	12
SHIRPA	Neurological	Females (males)	13, 66
Grip strength	Musculoskeletal/neurological	All	13, 66
Slit lamp/opthalmoscope	Vision	All	15, 49, 65, (73)
Optokinetic drum	Vision/neurological	All	15, 49, 65, (73)
Click box	Hearing	Non ahl*	14, 26, 39, 50
Auditory brainstem response+click stimulus	Hearing	Non ahl*	14, 39
Echo-MRI	Growth/body composition	Males (females)	16, 27, 51, 71
DEXA	Musculoskeletal/body composition	Females (males)	16, 51,
X-ray	Musculoskeletal	Females (males)	16, 51, 74
Pupillometry	Vision/neurobehaviour	All	18, 68
Sleep tracking	Neurobehaviour	Females (males)	18, 68
Clinical chemistry	Pathology	Females (males)	28, 53, 80
Fasted bleed	Diabetes/metabolism	Males (females)	17, 28, 52, 80
Fasted insulin	Diabetes/metabolism	Males (females)	33, 57, 72
IPGTT	Diabetes/metabolism	Males (females)	33, 57, 72

ECG, electrocardiogram; DEXA, dual energy X-ray analysis; IPGTT, intraperitoneal glucose tolerance test; MRI, magnetic resonance imaging; SHIRPA, SmithKline Beecham, Harwell, Imperial College and Royal London Hospital phenotype assessment.

A summary and timetable of the core phenotyping tests in the Harwell Ageing Screen, indicating screening time points. In addition to the documented phenotype tests mice are weighed every 3 months up until the age of 12 months and then monthly from 12 months onwards. For the tests where a single sex was initially tested the other sex was screened when outliers were suspected. The screening timetable is flexible and additional screens can be added to confirm outliers depending on the phenotype detected.

^*^G_3_ mice mothered by G_2_ females that do not carry the *Cdh23*^*ahl*^ allele.

**Table 2:** Summary of output from the ENU screen including number of pedigrees analysed along with mutant discovery and mutations cloned.

	**Total**	**Early**	**Late**			
Pedigrees	157	—	—			
Active pedigrees	23	—	—			
Pedigrees with phenotypes	72	58	26			
Number of mutants	105	78	27			
Mapped mutations	81	58	23			
Cloned mutations	44	32	12	Missense	Stop gained	Splice mutant
				19*/9	10/1	3/2

IAP, intracisternal A-particle.

One late and one early mutation result in an amino acid change but also affect splicing. Early phenotypes were defined as those identified in the first rounds of screening before 6 months of age, with late phenotypes being those detected after this time.

^*^Note that one mutation is the result of a spontaneous IAP insertion that appears to have occurred on the C3H background.

**Table 3:** Examples of phenotypes and mutations identified as part of the Harwell Ageing Screen.

**Pedigree**	**Phenotypic domain**	**Phenotype(s)**	**Age of detection (months)**	**Gene**	**CDS base change**	**Amino acid change**	**Supporting data**	**Current MGI alleles**	**Current MGI phenotypes**	**PubMed gene hits**
MPC-96	Deafness	Age-related hearing loss	⩾9	*Slc4a10*	1940T>C	L647P	a	1 targeted null	BehaviourGrowth/sizeHomeostasisMortality/agingNervous System	28
MPC-102	Neuro-degeneration	Gait abnormalities	⩾12	*Eftud1*	2948A>G	K983R	a	None	Glioma	4
MPC-151	Deafness	(a) Progressive hearing loss(b) Reduced fat mass	(a) 3(b) 7	*Wars2*	349G>T	V117L(+ splicing defects)	a, b	2 targeted null	Mitochondrial functionGWAS adiposity	7
MPC-173	VisionDeafness	(a) Deafness(b) Progressive corneal opacity	(a) ⩾3(b) ⩾9	*Ikzf2*	1551C>A	H517Q	a	2 targeted null	T-cell differentiationReduced growthIncreased mortalityAbnormal eyelid development	51
MPC-178	Cardiac	Hypertrophic cardiomyopathy	6	*Ecsit*	626G>T	N209I	a	1 targeted null	Embryonic lethal	28
MPC-200	SkinPathology	Epidermal and follicular hyperkeratosis	⩾12	*Ces2F*	1286A>T	Q429L	a	None	None	0
MPC-201	Vision	(a) Progressive reduction in visual acuity(b) Retinal degeneration	(a) ⩾12(b) ⩾12	*Idh3a*	685G>A	E229K	a	None	Retinitis Pigmentosa	29
MPC-205	Deafness	Progressive hearing loss	⩾6	*Ptprq*	5945+2T>C	Donor splice	a	4 targeted	Postnatal deafness	253
MPC-205	Renal Function	(a) Elevated creatinine/urea(b) Renal failure	(a) 6(b) 10–12	*Lama5*	2651A>G	E884G	a, b	One gene trapFour targeted	DevelopmentRenal FunctionHom null lethal	96
MPC-227	Body CompositionMusculo-skeletal	(a) Low-fat Mass(b) High-fat mass(c) Joint deterioration	(a) 3(b) 18(c) 15	*Acan*	5837C>T	A1946V	a	Three targeted null1 ENU inducedTwo spontaneous	CraniofacialGrowth/sizeAchondroplasia	4,246
MPC-236	Neuro-behaviourNeurological	(a) Sleep abnormalities(b) Motor function deterioration	(a) 4(b) 12	*Vamp2*	305T>A	I102N	a	Three targeted null	Synaptic vesicle functionGrowth/SizeMortality/AgeingHom null lethal	624
MPC-264	Deafness	Progressive hearing loss	⩾6	*Zfyve26*	3943C>T	R1315X	a	1 Endonuclease mediated	Spastic paraplegia	16

ENU, *N*-ethyl-*N*-nitrosourea.

The candidate gene, base change and amino acid change are indicated. Supplementary Information details whether (a) if this was the only medium or high-confidence coding mutation in the minimal mapping region and (b) where genetic complementation with a knockout allele has been carried out. We list the existing alleles documented on MGI along with the current phenotypic associations derived from MGI. The number of PubMed hits obtained by the gene name or synonyms is also provided. *Slc4a10*, ENSMUST00000112480; *Eftud1*, ENSMUST00000039881; *Wars2*, ENSMUST00000004343; *Ikzf2*, ENSMUST00000027146; *Ecsit*, ENSMUST00000180180*; Ces2F, ENSMUST00000076384; Idh3a, ENSMUST00000167866; Ptprq*, ENSMUST00000050702; *Lama5*, ENSMUST00000015791; *Acan*, ENSMUST00000032835; *Vamp2*, ENSMUST00000021273; and *Zfyve26*, ENSMUST00000021547. MGI, Mouse Genome Informatics. GWAS, Genome Wide Association Study. CDS, Coding DNA Sequence.
