# Supplementary material for: Novel gene function revealed by mouse mutagenesis screens for models of age-related disease
Source: Nat Commun. 2016 Aug 18;7:12444. doi: 10.1038/ncomms12444 (PMC4992138; doi:10.1038/ncomms12444)
Supplement: Supplementary Information — Supplementary Figures 1-5 and Supplementary Tables 1-3 [file ncomms12444-s1.pdf]

Figure S1

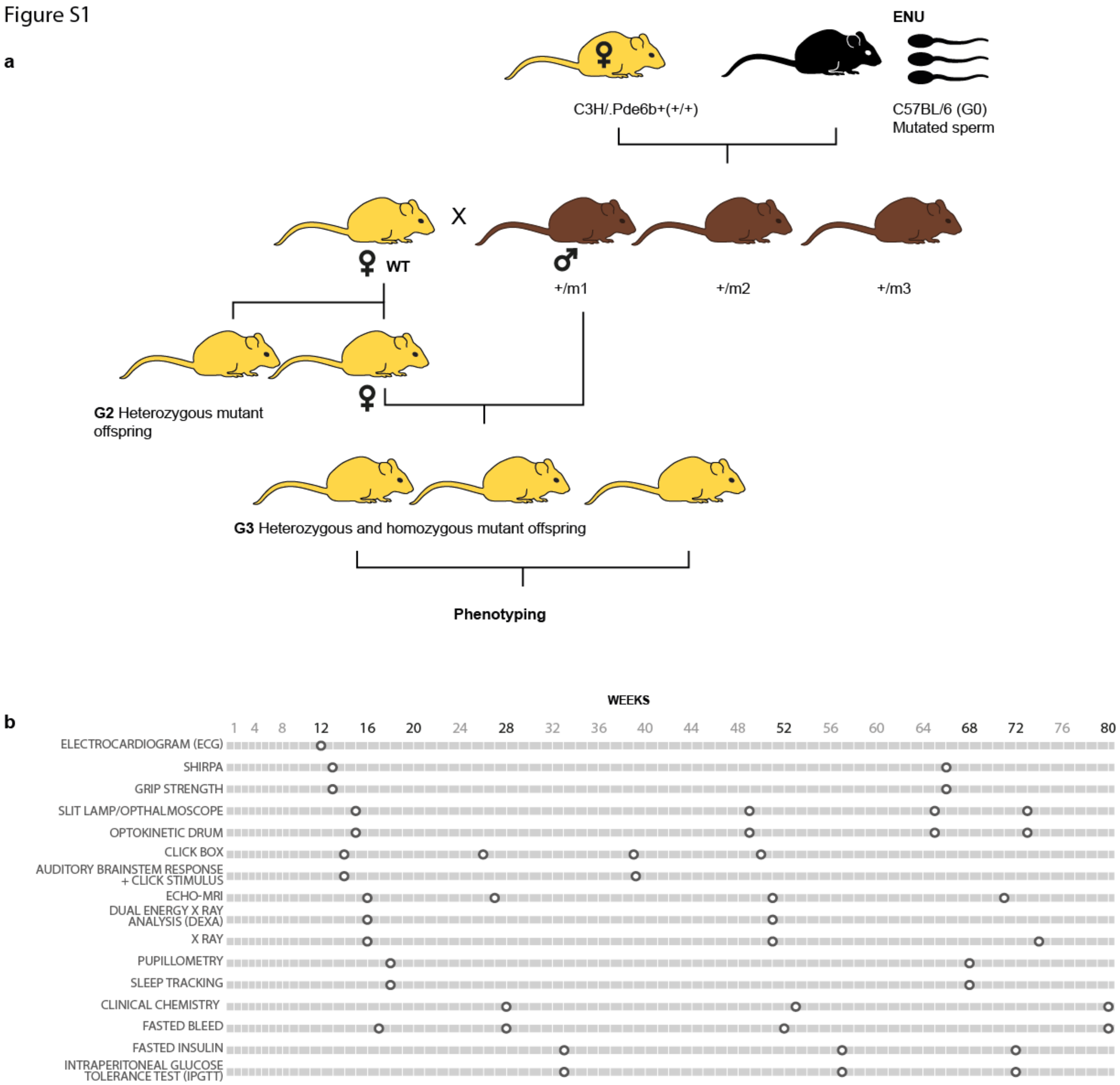
a
ENU
C3H/.Pde6b+(+/+)
C57BL/6 (G0)
Mutated sperm
X
WT
+/m1
+/m2
+/m3
G2 Heterozygous mutant offspring
G3 Heterozygous and homozygous mutant offspring
Phenotyping
b
WEEKS
1 4 8 12 16 20 24 28 32 36 40 44 48 52 56 60 64 68 72 76 80
ELECTROCARDIOGRAM (ECG)
SHIRPA
GRIP STRENGTH
SLIT LAMP/OPTHALMOSCOPE
OPTOKINETIC DRUM
CLICK BOX
AUDITORY BRAINSTEM RESPONSE + CLICK STIMULUS
ECHO-MRI
DUAL ENERGY X RAY ANALYSIS (DEXA)
X RAY
PUPILLOMETRY
SLEEP TRACKING
CLINICAL CHEMISTRY
FASTED BLEED
FASTED INSULIN
INTRAPERITONEAL GLUCOSE TOLERANCE TEST (IPGTT)

Supplementary Figure 1. An overview of the breeding and phenotyping in the Harwell Ageing Screen. (a) The Harwell Ageing Screen employed a G_3 breeding scheme illustrated here. A male G_0 (C57BL/6J) mouse is treated with ENU and crossed to wild-type female mice (C3H.Pde6b+) to produce G_1 offspring that are heterozygous for ENU-induced mutations. G_1 male mice are then outcrossed again to C3H.Pde6b+ females and G_2 daughters from this mating backcrossed to the G_1 father to generate G_3 progeny. Each G_1 male gives rise to a single pedigree of G_3 mice, which contain a range of mutations derived from the founder G_1 male. For each mutation an individual G_3 mouse may be homozygous, heterozygous or wild-type. **(b)** Mice were phenotyped at the time points outlined above and as described in Materials and Methods. The time points above are the core phenotyping pipeline with additional tests carried out where required.

Figure S2

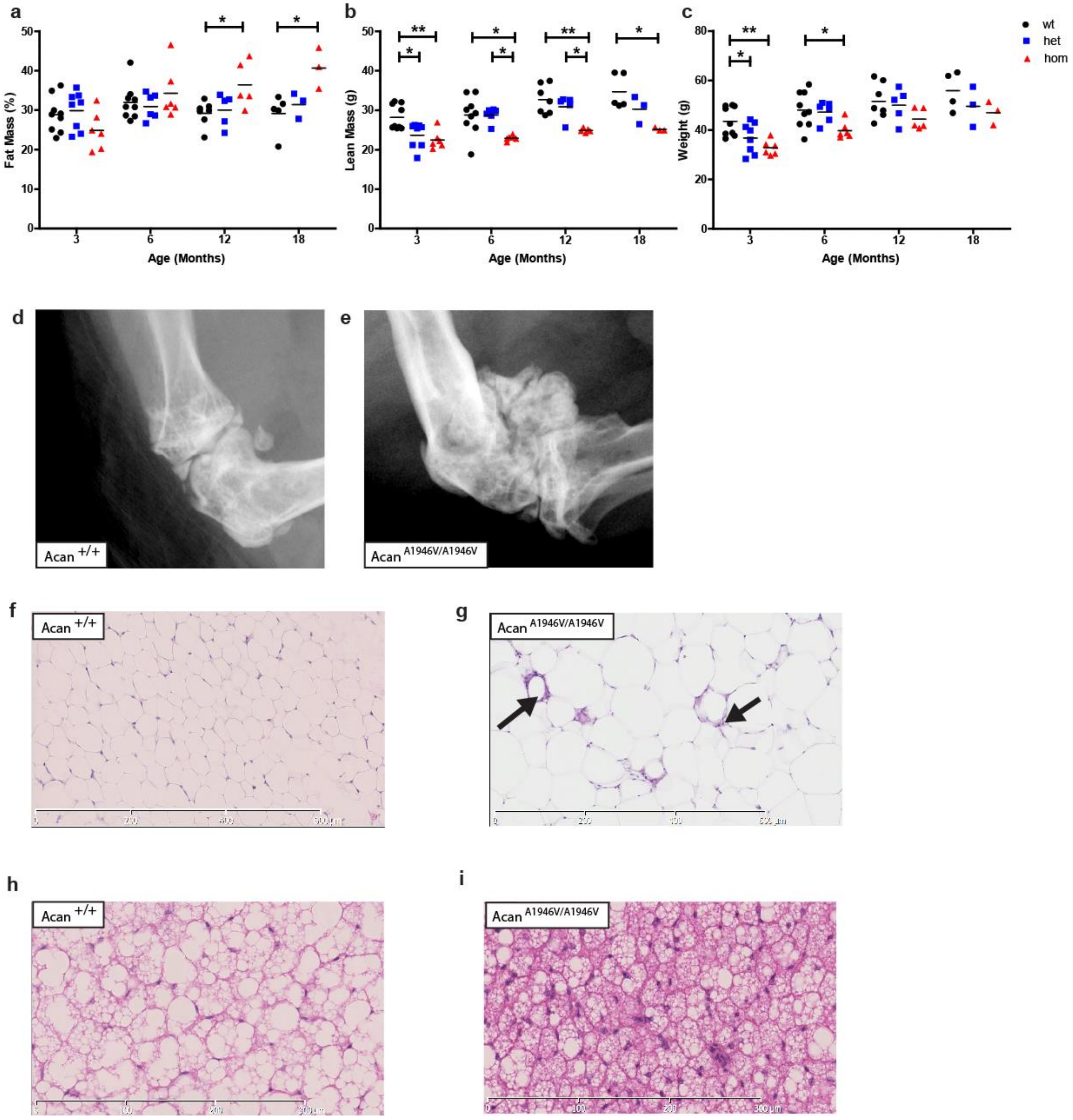

a
b
c
Fat Mass (%)
Lean Mass (g)
Weight (g)
Age (Months)
3
6
12
18
wt
het
hom
d
e
f
g
h
i
Acan +/+
Acan A1946V/A1946V

Supplementary Figure 2. Phenotyping of *Acan*^{A1946V} mutant mice. (a) Percentage fat mass of mice heterozygous (het) or homozygous (hom) for the *Acan*^{A1946V} mutation, and wild-type (wt) littermate controls from pedigree MPC-227, were determined by echo MRI. An increased percentage fat mass was observed in homozygous mutant mice at both 12 and 18 months compared to wild-type and heterozygous mice. **(b)** Homozygous *Acan*^{A1946V} mice have a significantly lower absolute lean mass at all ages tested when compared to wild-type animals. **(c)** From 6-months of age there were no significant differences in total bodyweight between mice heterozygous or homozygous for the *Acan*^{A1946V} mutation, or wild-type controls. Wild-type mice, n=8 at 3 months, n=8 at 6 months, n=7 at 12 months and n=4 at 18 months. Heterozygous mice n=7 at 3 months, n=6 at 6 months, n=5 at 12 months and n=3 at 18 months. Homozygous mice n=6 at 3 months, n=6 at 6 months, n=4 at 12 months and n=3 at 18 months. The mean is represented by a bar and significance determined using a one-way ANOVA with multiple comparisons with Tukey's multiple comparisons test comparing wild-type, heterozygote and homozygote mutant mice. X ray analysis of homozygous *Acan*^{A1946V} mice at 18 months of age showed bone deposition in several joints most significantly around knees and elbows. Control littermate knee **(d)** *Acan*^{A1946V} homozygote knee **(e)**. **(f) – (i)** Histological analysis of fat deposits also revealed qualitative differences in fat from *Acan*^{A1946V} homozygotes and wild-type littermates. Gonadal white adipose tissue from controls **(f)** or homozygous *Acan*^{A1946V} mice **(g)** show increased inflammatory infiltration (arrowed) and larger adipocytes. Analysis of brown adipose tissue showed increased fat accumulation in wild-type littermates **(h)** when compared to homozygous *Acan*^{A1946V} mice **(i)**.

Figure S3

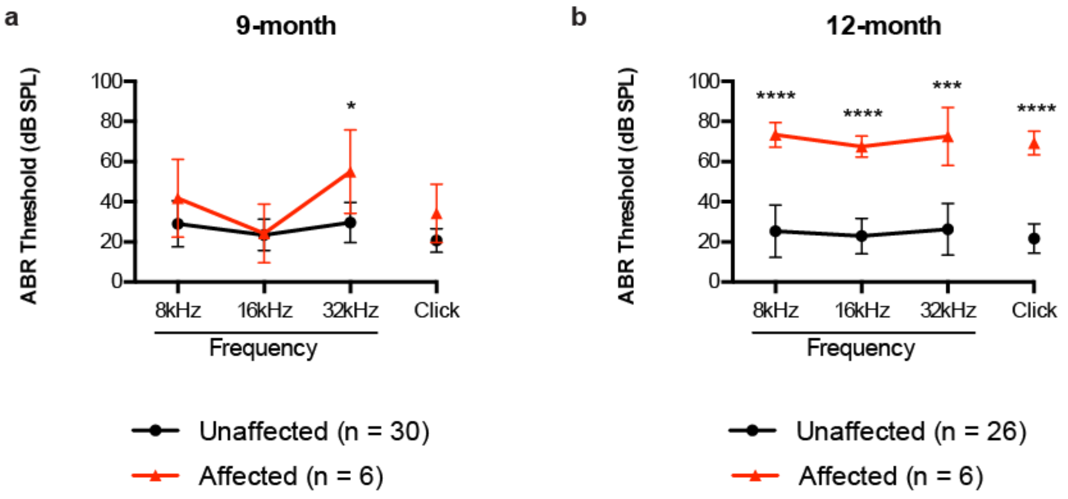

Supplementary Figure 3. Hearing data from MPC-96. Auditory thresholds for the G_3 mice from MPC-96 were determined using ABR phenotyping as part of the Harwell Ageing Screen. **(a)** At 9-months of age several mice showed mildly elevated hearing thresholds, particularly at 32 kHz. **(b)** Re-screening of these mice at an additional 12-month timepoint revealed their hearing impairment had progressed, with elevated hearing thresholds measured at all frequencies tested. Statistical significance was determined using a two-tailed unpaired t test with Welch's correction. * $P<0.05$; *** $P<0.001$.

Figure S4

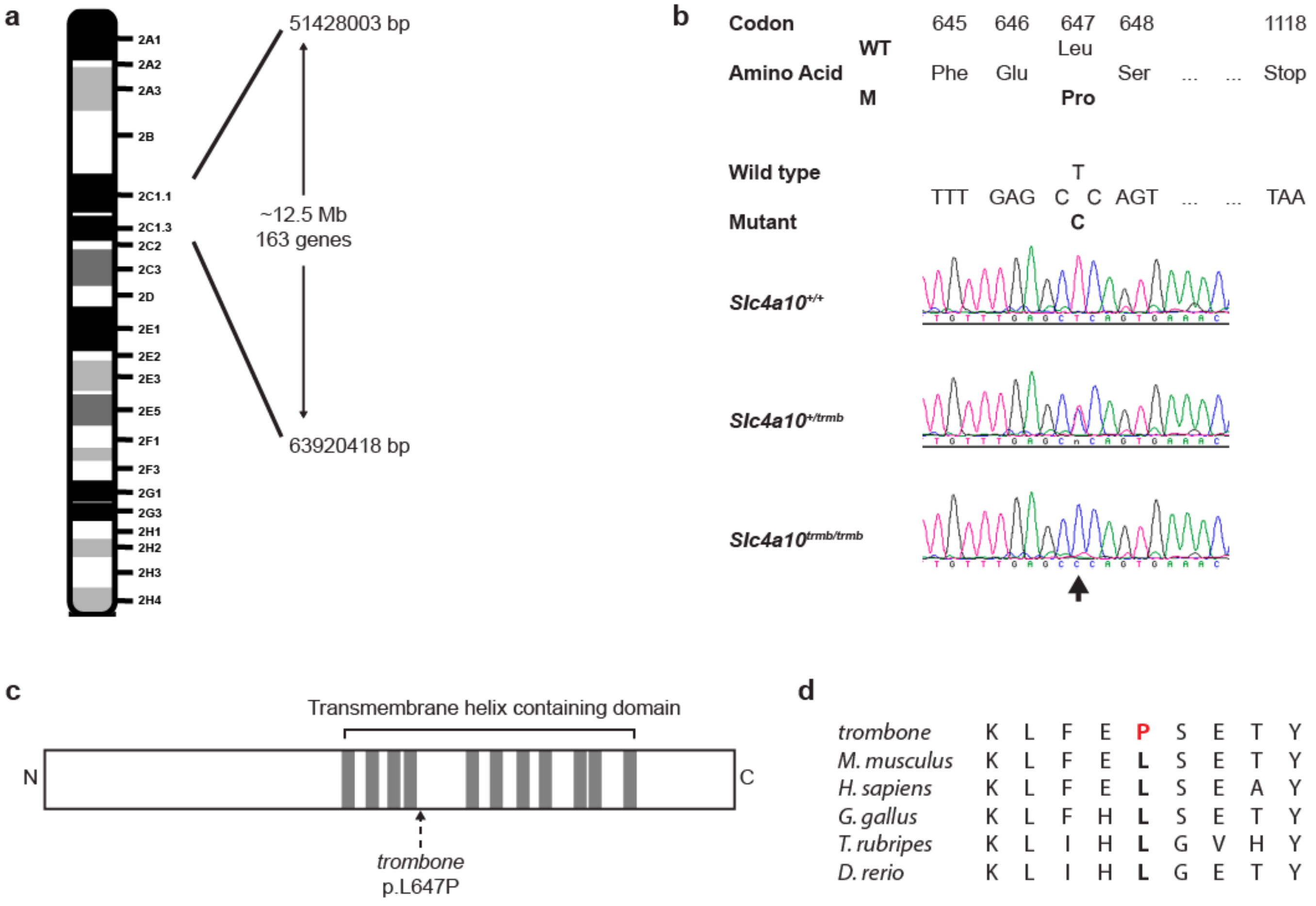

Supplementary Figure 4. Mapping and identification of the *trombone* mutation. (a) Diagram showing the location of the ~12.5 Mb critical interval identified for *trombone* on chromosome 2 (51572483-64082361 bp) (Genome assembly: GRCm38). **(b)** Confirmation of the NGS identified ENU-induced coding lesion in exon 15 of the *Slc4a10* gene (ENSMUST00000112480). The lesion consists of a nucleotide transversion (c.1940T>C) at codon 647, which alters the wild-type (WT) sequence C**T**C, encoding a Leucine (Leu, L), to the mutant (m) sequence C**C**C, encoding a Proline (Pro, P). Electropherograms showing the sequence surrounding *Slc4a10* nucleotide 1940 (indicated by an arrow) in normal hearing wild-type (*Slc4a10*$^{+/+}$) and heterozygous (*Slc4a10*$^{+/trmb}$) *trombone* mice and a hearing loss mutant (*Slc4a10*$^{trmb/trmb}$) mouse. **(c)** Schematic representation of the Slc4a10 protein illustrating the location of the mutation identified in *trombone*. The murine *Slc4a10* gene consists of 27 exons, spanning ~280 Kb of genomic DNA on

chromosome 2. Slc4a10 is a 1118 amino acid sodium bicarbonate cotransporter protein that contains many transmembrane domains (dark gray bars). The location of the homozygous Slc4a10 mutation identified in *trombone* by the present study is shown. **(d)** Evolutionary conservation of the Leucine (L) residue of Slc4a10 altered to Proline (P) in the *trombone* mutant. *H. Sapiens*, ENSG00000144290; *M. musculus*, ENSMUSG00000026904, *G. gallus*, ENSGALG00000001741; *T. rubripes*, ENSTRUG00000008287; *D. rerio*, ENSDARG00000063133.

Figure S5

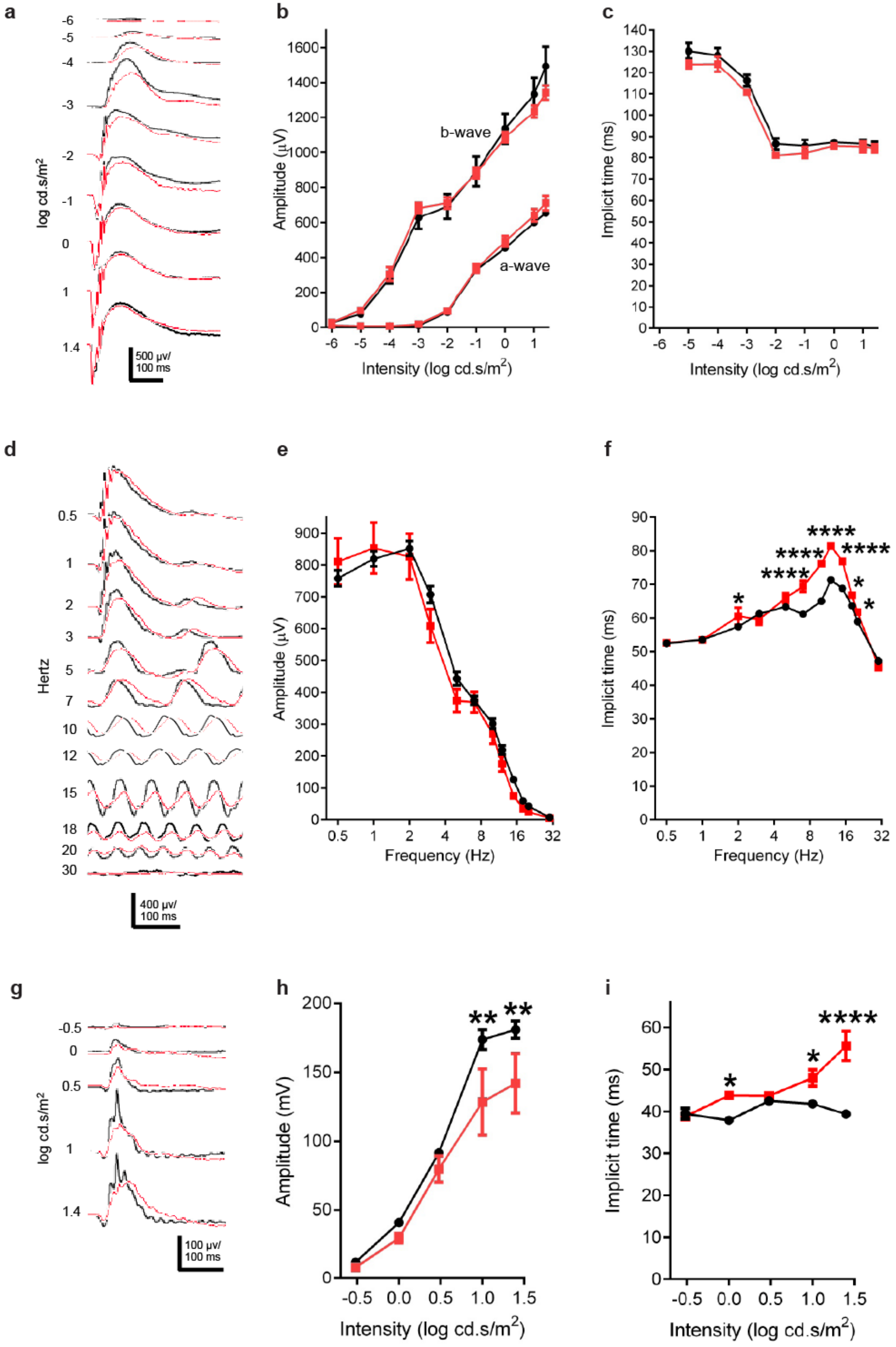
a
-6
-5
-4
-3
-2
-1
0
1
1.4
log cd.s/m2
500 µv/
100 ms
b
Amplitude (µV)
b-wave
a-wave
Intensity (log cd.s/m2)
c
Implicit time (ms)
Intensity (log cd.s/m2)
d
0.5
1
2
3
5
7
10
12
15
18
20
30
Hertz
400 µv/
100 ms
e
Amplitude (µV)
Frequency (Hz)
f
Implicit time (ms)
Frequency (Hz)
g
-0.5
0
0.5
1
1.4
log cd.s/m2
100 µv/
100 ms
h
Amplitude (mV)
Intensity (log cd.s/m2)
i
Implicit time (ms)
Intensity (log cd.s/m2)

Supplementary Figure 5. Electroretinography in *trombone* mice. (a) Representative traces from dark-adapted animals to single-flash stimuli of increasing stimuli (top to bottom, flash intensity shown on left margin in log cd.s/m^2). Responses of *Slc4a10*$^{trmb/trmb}$ mice (shown in red) are grossly similar to littermate, wild-type controls (in black). **(b)** Quantification of the amplitude (size) of a- and b-wave components in N=5 animals per group confirms that the size of responses is intensity dependent, but very similar between genotypes (Two-way repeated measures ANOVA for a-wave with intensity and genotype as factors, intensity P<0.0001, genotype P=0.4060, intensity x genotype P=0.6266. Two-way repeated measures ANOVA for a-wave with intensity and genotype as factors, intensity P<0.0001, genotype P= 0.7909, intensity x genotype P= 0.2054). **(c)** Quantification of the implicit time (speed) of the b-wave component in N=5 animals per group shows there is no significant difference in the timing of single-flash, dark-adapted recordings (Two-way repeated measures ANOVA with intensity and genotype as factors, intensity P<0.0001, genotype P= 0.3375, intensity x genotype P= 0.5354). **(d)** Representative traces from dark-adapted animals to flickering stimuli of a fixed intensity (-2 log cd.s/m^2) of increasing frequency (top to bottom, frequency shown on left margin in Hertz). The responses of *Slc4a10*$^{trmb/trmb}$ mice (show in red) are grossly similar to littermate, wild-type controls (in black) at lower frequencies (0.5 - 5 Hz) but differences in the phase/timing of responses become apparent to faster flicker (10 – 15 Hz). **(e)** Quantification of the peak-to-peak amplitude of the waveforms in N=5 animals per group shows the size of the wave changes as a function of stimulus frequency but is not different between genotypes (Two-way repeated measures ANOVA with frequency and genotype as factors, frequency P<0.0001, genotype P= 0.6266, intensity x genotype P= 0.2324). **(f)** Quantification of the delay until the first positive deflection (implicit time) of the waveforms in N=5 animals per group shows the timing of responses is dependent on the stimulus frequency and this relationship is very different between genotypes (Two-way repeated measures ANOVA with frequency and genotype as factors, frequency P<0.0001, genotype P= 0.2123, intensity x genotype P<0.0001). Pairwise comparisons show that responses of mutants are very significant delayed compared to wild-types principally in the 7 -15 Hz range. **(g)** Representative traces from light-adapted animals to single-flash stimuli of increasing stimuli (top to bottom, flash intensity shown on left margin in log cd.s/m^2). Responses to dimmer stimuli are small and similar between genotypes but responses to higher flash intensities appear to be much smaller and slower in *trombone* mice (in red) compared to littermate, wild-type controls (in black). **(h)** Quantification of the amplitude (size) of the b-wave in N=5 animals per group confirms that the size of responses is intensity dependent but that responses to higher flash intensities are significantly smaller in mutants than wild-types (Two-way repeated measures ANOVA with intensity and genotype as factors, intensity P<0.0001, genotype P= 0.1174, intensity x genotype P= 0.0251). **(i)** Quantification of the implicit time of the b-wave in N=5 animals per group shows responses are significantly slower in *trombone* mice compared to littermate, wild-type controls (Two-way repeated measures ANOVA with intensity and genotype as factors, intensity P= 0.0004, genotype P= 0.0061, intensity x genotype P<0.0001). In all panels *Slc4a10*$^{trmb/trmb}$ mice are show in red and littermate, wild-type controls are indicated in black. In all graphs plotted values are mean$\pm$SEM, N=5. The following symbols indicate significant pairwise comparisons in Bonferroni's multiple comparisons test: * P≤0.05, ** P≤0.01, **** P≤0.0001.

Ageing Screen Procedure	Ageing IMPRESS identifier[1]	Equivalent IMPC procedure[2]	False Positive Rate[3]
**Intraperitoneal glucose tolerance test**	**IMPC_IPG_001**	**IMPC_IPG_001**	0.64%
**Body Composition (DEXA lean/fat)**	**IMPC_DXA_001**	**IMPC_DXA_001**	0.62%
**Electrocardiogram**	**IMPC_ECG_001**	**IMPC_ECG_001**	0.64%
**Electrocardiogram**	**IMPC_ECH_001**	**IMPC_ECH_001**	0.61%
**Auditory brainstem response**	**IMPC_ABR_002**	**IMPC_ABR_002**	0.92%
**Hematology**	**IMPC_HEM_002**	**IMPC_HEM_002**	0.63%
**FACs Analysis**	**IMPC_ACS_003**	**IMPC_ACS_003**	0.60%
**Clinical Blood Chemistry**	**IMPC_CBC_003**	**IMPC_CBC_003**	0.64%
**SHIRPA**	**IMPC_CSD_003**	**IMPC_CSD_003**	1.22%
**Indirect Calorimetry**	**IMPC_CAL_003**	**IMPC_CAL_003**	0.58%
		**Overall:**	0.71%

Supplementary Table 1. Estimation of the false positive rate of the reference range phenotype detection method. Phenotype procedures that occur in both the IMPC project and the Harwell ageing screen were selected for the FPR analysis. [1]Ageing IMPRESS identifier: documents the IMPRESS identifiers for the ageing procedure used. [2]Equivalent IMPC procedure: documents the equivalent IMPC IMPRESS identifier. Reference ranges were created using IMPC wildtype animals for each parameter in the procedures. [3]False positive rate: FPRs determined from the reference range method as described in Methods.

Pedigree ID	Phenotype Description	Chr	Gene	Category	CDS position	Mutation	Functional Class	SIFT Score	Supporting Evidence	Novelty	Known Gene-Phenotype association
**MPC-59**	Yellow coat colour	8	*Mcr1*	Early	N/A		IAP insertion	N/A	s, a, f, e, p	Known gene - Known function	Coat Colour
**MPC-63**	Progressive tremors from 9 months	7		Late							
**MPC-66**	Abnormal gait from 4 months	18		Early							
**MPC-81**	Reduced fat mass	8		Late							
**MPC-81**	Coat colour			Early							
**MPC-87**	Hydronephrosis, tubular dilation and vacuolation			Early							
**MPC-91**	Sudden death	7	*Bcat2*	Early	988C>T	Q330*	Stop Gain	NP	s, f	Known gene – Novel function	Maple Syrup Urine Disease
**MPC-91**	Hyperactivity, impaired hearing	4	*Whrn*	Early	1068+1C>T	Intronic	Splice Donor	NP	s, g, e, p	Known gene - Novel function	Deafness/ Retinal/Schizophrenia
**MP-95**	Ataxia			Early							
**MPC-96**	Low HDL and total LDL	10	*Pla2g12b*	Early	196T>A	Y66N	Missense	Damaging (0.006)	s, e, p	Known gene - Known function	Cholesterol transport/fatty liver
**MPC-96**	Reduced body size	12	*Pld4*	Early	472C>T	L158F	Missense	Damaging (0.008)	s, p	Known gene - Known function	Growth/size
**MPC-96**	Late onset and progressive hearing loss	2	*Slc4a10*	Late	1940T>C	L647P	Missense	Damaging (0.007)	s, f, e	Novel gene - Novel function	Retinal dysfunction
**MPC-96**	Cataracts			Late							
**MP-97**	Situs Inversus	15		Early							
**MPC-102**	Abnormal gait from 12 months, progressive deterioration	7	*Eftud1*	Late	2948A>G	K983R	Missense	Tolerated (0.607)	s, f, e	Known gene - Novel function	Ribosomal maturation
**MPC-107**	Elevated creatinine and urea at 18 months			Late							
**MP-107**	X ray abnormalities identified at 4 months with late onset joint degeneration	11		Late							
**MPC-111**	Low BMD and body weight	1		Early							
**MPC-116**	X Ray abnormalities on knees			Late							
**MPC-119**	Testicular calcification at 18 months			Late							
**MPC-119**	Low fat mass			Early							
**MPC-121**	Tremors/low grip strength	3		Early							
**MPC-125**	Low bone mineral density, low fat mass, low body weight	4	*Lpar1*	Early	N/A	N/A	Intronic	N/A	s	Known gene – Novel function	Bone growth

**MPC-131**	Elevated ALP, ALT, AST, reduced inorganic phosphate and albumin. Reduced body weight	2		Early							
**MPC-134**	Elevated AST	16		Early							
**MPC-142**	Deafness with vestibular defects	7	*Myo7a*	Early	1515C>A	N505K	Missense	Damaging (0.01)	a	Known gene – Known function	Deafness with vestibular defects
**MPC-151**	Progressive hearing loss, reduced fat mass, cardiomyopathy	3	*Wars2*	Late	349G>T	V117L	Missense and splice	Damaging (0.013)	s, g, f, e, p	Novel gene – Novel function	GWAS Waist-Hip ratio
**MPC-162**	Reduced visual acuity			Early							
**MPC-165**	Impaired glucose tolerance			Early							
**MPC-168**	Impaired glucose tolerance			Early							
**MPC-169**	Deafness with vestibular defects	5		Early							
**MPC-172**	Impaired glucose tolerance			Early							
**MPC-173**	Deafness/ Progressive corneal opacity	1	*Ikzf2*	Early/Late	1551C>A	H517Q	Missense	Damaging (0.00)	s, f, e	Novel gene – Novel function	T cell development
**MPC-174**	Coat colour	7		Early							
**MPC-178**	Hypertrophic cardiomyopathy	9	*Ecsit*	Late	626A>T	N209I	Missense	Damaging (0.008)	s, f, e, p	Novel gene – Novel function	Mitochodrial complex I assembly, TLR signal transduction
**MPC-178**	Reduced bone mineral density, reduced growth	1	*Irs1*	Early	655G>T	E219*	Stop Gain	NP	s, e, p, a	Known gene – Known function	Insulin signalling
**MPC-178**	Low fat and lean mass	7	*Herc2*	Early	13476T>A	C4492*	Stop Gain	NP	s, p, a	Known gene – Known function	Prader Willi Sydrome
**MPC-184**	Reduced fat mass	13		Early							
**MPC-185**	Reduced fat mass	3		Early							
**MPC-186**	Fatty liver	2		Late							
**MPC-187**	Fitting and hyperactivity	11	*Ap2b1*	Early	16T>A	Y6N	Missense	Damaging (0.00)	s	Known gene – Novel function	Clathrin endocytosis
**MPC-188**	Deafness	13	*Gpr98*	Early	8554+2T>C	Donor splice	Intronic	NP	s, e, p, a	Known gene – Known function	Deafness
**MPC-188**	Deafness	18	*Loxhd1*	Early	4370A>T<br>5323G>A	I1457N<br>T1775A	Missense<br>Missense	Damaging (0.001)<br>Tolerated (0.14)	s, e, p, a	Known gene – Known function	Deafness
**MPC-190**	Impaired glucose tolerance			Early							
**MPC-190**	Deafness	11	*Myo15*	Early	4940A>G	D1647G	Missense	Damaging (0.001)	s, f, e, p, a	Known gene –	Deafness

										Known function	
**MPC-191**	Progressive Tremors	13	*HexB*	Early	675T>G	Y225*	Stop Gain	NP	s, f, e, p, a	Known gene – Known function	Sandhoff syndrome
**MPC-200**	Epidermal and follicular hyperkeratosis	8	*Ces2F*	Late	1286A>T	Q429L	Missense	Tolerated (0.3)	s	Novel gene – Novel function	Carboxylic ester hydrolase activity
**MPC-201**	Impaired glucose tolerance			Early							
**MPC-201**	Progressive retinal degeneration and reduced visual acuity from 12 months	9	*Idh3a*	Late	685G>A	E229K	Missense	Damaging (0.028)	s, e, p	Known gene-known function	Retinitis pigmentosa
**MPC-202**	Age-related hearing loss	2		Late							
**MPC-203**	Age-related hearing loss	1		Late							
**MPC-203**	Tremors and abnormal gait			Early							
**MPC-205**	Elevated creatinine and urea from 6 months	2	*Lama5*	Late	2651A>G	E884G	Missense	NP	s, g, e, p	Known gene -Novel function	Organogenesis
**MPC-205**	Age-related hearing loss	10	*Ptprq*	Late	5945+2T>C	Intronic	Splice Donor	NP	s, , e, p, a	Known gene -Novel function	Early onset deafness
**MPC-214**	Decreased pupillary response	13	*Chrm3*	Early	35T>A	L12*	Stop Gain	NP	s, e, f, p	Known gene – Known function	Muscle function
**MPC-225**	Impaired glucose tolerance			Early							
**MPC-225**	Abnormal gait	7	*Mag*	Early	328G>T	E110*	Stop Gain	NP	s, e, p, a	Known gene – Known function	Axonal function
**MPC-227**	Impaired glucose tolerance			Early							
**MPC-227**	Low cholesterol	4	*Abca1*	Early	1196T>A	V399E	Missense	Damaging (0.009)	s, e, p, a	Known gene – Known function	Tangiers disease
**MPC-227**	Obesity and joint degeneration	7	*Acan*	Late	5837C>T	A1946V	Missense	Damaging (0.00)	s, e, p	Known gene -Novel function	Bone/Growth, osteoarthritis
**MPC-227**	Deafness	2		Early	Multiple	Non-Coding					
**MPC-231**	High fasted glucose	11		Early							
**MPC-231**	Polycystic Kidneys			Early							
**MPC-231**	Deafness	18	*Loxhd1*	Early	5087C>T	T1696M	Missense	Damaging (0.00)	s, , e, p, a	Known gene – Known function	Deafness
**MPC-231**	Deafness	18	*Hars*	Early	331T>C	S111P	Missense	Damaging (0.011)	s	Novel gene – Novel function	Histidyl-tRNA synthetase

**MPC-232**	Elevated ALT	6	*Trim24*	Early	714T>A	C238*	Stop Gain	NP	s, e, p, a	Known gene – Known function	Cell cycle control
**MPC-232**	Renal developmental abnormalities	8	*PskH1*	Early	23T>A	V8D	Missense	Damaging (0.001)	s, f, e		
**MPC-233**	Reduced body size, hyperactivity			Early							
**MPC-234**	High fasted glucose and fructosamine			Late							
**MPC-234**	Deafness	5	*Slc26a5*	Early	1136G>T	G379V	Missense	Damaging (0.00)	s, , e, p, a	Known gene – Known function	Deafness
**MPC-234**	Progressive hearing loss	8	*Nek5*	Early	1660G>A	A554T	Missense	Tolerated (0.56)	s	Known gene – Novel Function	Skeletal muscle differentiation
**MPC-236**	Decreased sleep, late motor function deterioration	11	*Vamp2*	Late	305T>A	I102N	Missense	Damaging (0.00)	s, f, e, a	Known gene – Novel Function	Synaptic vesicle docking
**MPC-242**	Impaired glucose tolerance, insulin resistance, obesity, diarrhoea	13	*Pcsk1*	Early	286G>T	V96L	Missense	Damaging (0.01)	s, f, e, p, a	Known gene – Known function	Human mutations and GWAS Obesity
**MPC-244**	Neonatal malaise, ataxia	12		Early							
**MPC-246**	Deafness and vestibular defects	18	*Slc12a2*	Early	1728T>A	C576*	Stop Gain	NP	s, e, p, a	Known gene-Known function	Deafness and vestibular defects
**MPC-253**	Colitis	13		Late							
**MPC-256**	Elevated Creatinine and urea at 12 months	5		Late							
**MPC-264**	Progressive hearing loss and tremors	12	*Zfyve26*	Late	3943C>T	R1315*	Stop Gain	NP	s, e, p	Known gene -Novel function	Spastic paraplegia 15
**MPC-264**	Deafness	13	*Slc12a7*	Early	1795C>T	Q599*	Stop Gain	NP	s, e, p, a	Known gene-Known function	Deafness and renal tubular acidosis
**MPC-265**	Deafness and vestibular defects	5	*Grxcr1*	Early	552C>A	N184K	Missense	Tolerated (0.089)	s, e, p, a	Known gene-Known function	Deafness
**MPC-265**	Deafness	19	*Pdzd7*	Early	833T>C	L278P	Missense	Damaging (0.00)	s, e, p, a	Known gene-Known function	Deafness
**MPC-267**	Increased sleep	3		Early							
**MPC-269**	Retinal degeneration	14	*Rpgrip1*	Early	N/A	N/A	Intronic	NP	s, e, p, a	Known gene-Known function	Leber congenital amaurosis
**MPC-269**	Progressive hearing loss	7	*Tmem145*	Early	147T>A	C49*	Stop Gain	NP	s	Novel gene – Novel Function	G-protein coupled receptor signalling
**MPC-274**	Coat colour			Early							
**MPC-275**	Tail kink	6		Early							
**MPC-276**	Increased sleep	1		Early							

**MPC-282**	Impaired glucose tolerance/glycosouria	13		Early							
**MPC-285**	Impaired glucose tolerance	7		Early							
**MPC-285**	Deafness and vestibular defects	9	*Myo6*	Early	1382-2A>G	Intronic	Splice Acceptor	NP	s, e, p, a	Known gene-Known function	Deafness and vestibular defects
**MPC-285**	Decreased pupillary response	6		Early							
**MPC-286**	Impaired glucose tolerance	12		Late							
**MPC-286**	Reduced fat mass			Early							
**MPC-290**	Craniofacial abnormalities	10		Early							
**MPC-290**	Low fat and lean mass	11		Early							
**MPC-290**	Deafness	17		Early							
**MPC-290**	Deafness	2		Early							
**MPC-291**	Impaired glucose tolerance			Early							
**MPC-292**	Limb grasp and progressive tremors	7		Late							
**MPC-294**	Obesity	14	*Gnrh1*	Early	73T>C	S25P	Missense	Damaging (0.00)	s, e, p, a	Known gene-Known function	Endocrine/exocrine function, growth
**MPC-295**	Neonatal jaundice	1		Early							
**MPC-298**	Retinal degeneration	1		Late							
**MPC-303**	Limb grasping	2		Early							
**MPC-312**	Deafness and vestibular defects			Early							
**MPC-312**	Impaired glucose tolerance	2		Early							

Supplementary Table 2 - An overview of the current output of the Harwell Ageing Screen listing map locations and genes containing the causative mutation where known.

A phenotypic description for each mutant identified as part of the Harwell Ageing Screen is shown above with key details of the mutation. The chromosomal location of the causative allele is listed and where possible the gene. Mutants are classified as early or late according the time point the phenotype was originally identified in the screening pipeline; early being before and late being detected after 6 months of age. Pedigrees with Late onset mutations are highlighted. We show the supporting evidence for the role of the listed mutation in the development of the observed phenotype; s = confirmation of the mutation through Sanger sequencing of affected individuals, g= genetic proof through complementation studies, f= a relevant functional deficit or alteration in the protein, e=expression of the protein is within relevant tissue(s), p=the phenotype relevant to known function of gene, and a = the existence of additional mouse alleles with very similar phenotypes. The coding DNA sequence (CDS) position affected, amino acid

change and SIFT scores are given where possible (NP = no prediction from SIFT analysis). For mutants where the gene has been identified, we have highlighted two classes: 1) known gene – novel function: loci were the gene already has well described functions but the mutant reveals novel functionality 2) novel gene – novel function: loci where no function has been ascribed to date to the gene in the phenotypic area under investigation, and where novel function is revealed through the mutant phenotype. For both cases, the known gene-disease associations are briefly described.

Supplementary Table 3. List of ENU-induced SNVs within the *trombone* mapped critical interval (Chr2:51572483-64082361, GRCm38).

Position	Reference allele	Alternative allele	Functional Class	Entrez Gene Name	Transcript SNV Position	Amino Acid Position	Amino Acid Reference	Amino Acid Alternative	Read Depth	Alternative allele frequency
**52274671**	G	A	Intron variant	*Neb*	.	.	.	.	19	12
**52295606**	A	G	Intron variant	*Neb*	.	.	.	.	32	19
**52368209**	G	A	Intergenic variant	.	.	.	.	.	15	6
**52610937**	A	G	Intron variant	*Cacnb4*	.	.	.	.	14	6
**52830164**	T	C	Intergenic variant	.	.	.	.	.	13	6
**53081955**	A	G	Intron variant	*Fmnl2*	.	.	.	.	23	10
**53537814**	A	G	Intergenic variant	.	.	.	.	.	12	5
**53698132**	T	C	Upstream gene variant	.	.	.	.	.	23	11
**54032394**	C	G	Intergenic variant	.	.	.	.	.	13	8
**54046845**	A	G	Intergenic variant	.	.	.	.	.	19	10
**55371519**	T	C	Intergenic variant	.	.	.	.	.	22	7
**55449695**	T	A	Intron variant	*Kcnj3*	.	.	.	.	24	11
**55707875**	T	A	Intergenic variant	.	.	.	.	.	28	14
**55922589**	G	A	Intergenic variant	.	.	.	.	.	14	6
**56207939**	T	A	Intergenic variant	.	.	.	.	.	13	5
**56505251**	T	C	Intergenic variant	.	.	.	.	.	15	8
**57344733**	T	A	Intron variant	*Gpd2*	.	.	.	.	18	8
**57754165**	A	G	Intergenic variant	.	.	.	.	.	19	12
**58028598**	A	G	Intron variant	*Galnt5*	.	.	.	.	22	11
**58263200**	G	A	Downstream gene variant	*Acvr1c*	.	.	.	.	12	7
**59389135**	G	A	Upstream gene variant	.	.	.	.	.	34	17
**59964245**	A	T	Intron variant	*Baz2b*	.	.	.	.	24	11
**60775938**	A	T	Intron variant	*Rbms1*	.	.	.	.	22	11
**60930362**	T	C	Intron variant	*Rbms1*	.	.	.	.	30	21
**61624059**	T	A	Intron variant	*Tank*	.	.	.	.	24	13
**61685164**	C	T	Intergenic variant	.	.	.	.	.	17	9
**61872836**	T	C	Intergenic variant	.	.	.	.	.	23	7
**62268849**	T	C	Missense variant	*Slc4a10*	1940	647	L	P	19	12
**62654908**	C	T	Intergenic variant	.	.	.	.	.	21	11
**62906040**	A	T	Intron variant	*Kcnh7*	.	.	.	.	17	11
**62928313**	T	C	Intron variant	*Kcnh7*	.	.	.	.	22	13

**62948024**	T	C	Intron variant	*Kcnh7*	.	.	.	.	21	10
**63048732**	A	T	Intron variant	*Kcnh7*	.	.	.	.	15	7
**63508788**	C	T	Intergenic variant	.	.	.	.	.	24	11
**64023704**	A	G	Intron variant	*Fign*	.	.	.	.	19	11
